# Exploring the ethnobiological practices of fire in three natural regions of Ecuador, through the integration of traditional knowledge and scientific approaches

**DOI:** 10.1186/s13002-024-00699-4

**Published:** 2024-06-06

**Authors:** Vinicio Carrión-Paladines, Liliana Correa-Quezada, Huayra Valdiviezo Malo, Jonathan Zurita Ruáles, Allison Pereddo Tumbaco, Marcos Zambrano Pisco, Nataly Lucio Panchi, Leticia Jiménez Álvarez, Ángel Benítez, Julia Loján-Córdova

**Affiliations:** 1 Departamento de Ciencias Biológicas y Agropecuarias, Universidad Técnica Particular de Loja, San Cayetano Alto S/N, C.P. 11 01 608, Loja, Ecuador; 2 Departamento de Ciencias Jurídicas, Universidad Técnica Particular de Loja, San Cayetano Alto S/N, Loja, 1101608 Ecuador; 3 Licenciatura en Gestión Ambiental, Universidad Técnica Particular de Loja, San Cayetano Alto S/N, Loja, 1101608 Ecuador

**Keywords:** Regions of Ecuador, Traditional use of fire, Fire weather, Fire severity, Integrated fire management strategies

## Abstract

This study examines the convergence between traditional and scientific knowledge regarding the use of fire and its potential to trigger wildfires, with possible impacts on ecosystems and human well-being. The research encompasses three distinct natural regions of Ecuador: the coast, the highlands, and the Amazon. Data on traditional fire use were collected through semi-structured interviews with 791 members from five local communities. These data were compared with climatic variables (rainfall (mm), relative humidity (%), wind speed (km/h), and wind direction) to understand the climatic conditions conducive to wildfires and their relationship with human perceptions. Furthermore, the severity of fires over the past 4 years (2019–2022) was assessed using remote sensing methods, employing the Normalized Burn Ratio (NBR) and the difference between pre-fire and post-fire conditions (NBR Pre-fire–NBR Post-fire). The results revealed a significant alignment between traditional knowledge, climatic data, and many fires, which were of low severity, suggesting potential benefits for ecosystems. These findings not only enable the identification of optimal techniques and timing for traditional burns but also contribute to human well-being by maintaining a harmonious balance between communities and their environment. Additionally, they provide valuable insights for the development of more inclusive and effective integrated fire management strategies in these natural areas of Ecuador.

## Introduction

Historical ecology and ethnobiology provide robust evidence of the significant role human activities have played in ecological change over time, challenging the prevailing notion that such changes were solely due to natural and climatic processes [1–3]. Extensive research indicates that, over millennia, ecosystem changes have been primarily driven by human actions, which have profoundly altered the structure and functioning of ecosystems [4]. Among these human activities, the use of fire stands out as a long-standing traditional practice, likely persisting for thousands of years, and has been a widely employed tool for environmental transformation by human societies [5]. This historical fire regime, established over long periods, has exerted a direct and substantial influence on plant composition and other life forms [6].

Ethnographic records provide numerous examples of landscape management strategies involving burning, particularly notable in Australia and North America [7]. However, this pattern is also evident in many other regions of the world, including South America. For instance, recent research in South America suggests that indigenous land use and traditional burning practices have significantly shaped the floristic composition and forest structure of the Amazon over millennia, especially during the peak of pre-Columbian indigenous occupation [8]. Thus, understanding historical ecology and ethnobiology leads us to recognize the crucial role of human activities in shaping ecosystems over time. These ancestral practices, such as the use of fire, have left an indelible mark on vegetation and biodiversity, underscoring the importance of comprehending, and conserving traditional landscape management techniques to ensure the sustainability of natural resources. Given this context, it is essential to study the relationship between local knowledge and scientific knowledge regarding the use of fire. Such an approach not only enriches our understanding of historical and contemporary fire regimes but also highlights the value of integrating traditional ecological knowledge with scientific data. Recognizing that local knowledge about the use of fire is as important as scientific knowledge helps to preserve these ancient practices and promotes a more holistic and sustainable approach to ecosystem management.

A review of ethnobiology in Latin America has shed light on the current state of ethnobiological research in the region [9]. Countries like Brazil and Mexico appear to be leaders in the field, whereas in Ecuador, there seems to be no research in this area [10]. Ecuador ranks last in Latin America in terms of scientific production specifically related to ethnobiology. Alburquerque et al. [9] demonstrate that from 1963 to 2012, only one Ecuadorian scientific article has been published in the entire field, compared to 289 in Brazil, 153 in Mexico, 61 in Peru, and 11 in Colombia. Therefore, this reality presents an opportunity to initiate such studies, particularly around human use of fire, as the country boasts 13 different indigenous nationalities, each with its own language, history, and culture, along with a large mestizo population, providing a self-sufficient framework for interaction with nature [11].

In the aforementioned framework, cultural burning emerges as one of the most powerful tools used by humans to transform landscapes [12, 13]. It has been employed for various purposes, such as land clearing for the creation of public, domestic, and agricultural spaces, as well as for slash-and-burn farming [14], food preparation, and waste disposal [15]. Additionally, charcoal produced through cultural burning has been shown to enhance soil fertility and contribute to the formation of anthropogenically modified soils, such as Amazonian Dark Earths and Amazonian Brown Earths [16]. Moreover, for instance, indigenous communities in North America manipulated their environment to favor plants and animals necessary for their subsistence, shelter, clothing, and other vital needs [17]. Furthermore, traditional communities extensively use fire in other activities, including hunting, crop enhancement, pest control, habitat diversification, pasture management, large-scale fire prevention, wood gathering, travel route maintenance, riparian area cleaning, basketry material cultivation, communication, and ceremonies [18, 19]. Huffman [20] observed that these communities possess knowledge about fire effects on fungi, plants, and animals, emphasizing appropriate burning timing considering plant phenology, season, fuel moisture, time since the last fire (and its severity), and fire behavior. Rojas Rabiela [21] noted the persistent use of the slash-and-burn system in various regions, involving machete cutting of large trees, bushes, grasslands, and vines. This system accumulates biomass through burning, and on the resulting ash layer, traditional farmers grow food crops such as maize (*Zea mays* L.) and beans (*Phaseolus vulgaris* L.) for human consumption [22]. This shaping of interactions between humans and nature developed over millennia has had a significant impact on the distribution of plant species, the expansion of grasslands, and the configuration of forests [4].

On the other hand, recent studies, such as those by Lake et al. [23] and Roos et al. [24], highlight the importance of fire use in Native American cultures. For instance, in Canada, traditional fire management by indigenous communities has been shown not only to promote ecosystem diversity but also to facilitate the management of complex resources [25, 26]. Additionally, these practices help reduce the risk of wildfires by decreasing fuel loads. These findings underscore how biodiversity conservation is inherently linked to fire management strategies implemented by indigenous communities. In South America, communities such as the Mebêngokrê in Brazil and the Pemón in Venezuela incorporate fire into cultural practices, influencing social processes and knowledge transmission [14, 27]. Similarly, mestizo communities, like those in the Chiapas Biosphere Reserve [28], also utilize local fire knowledge. This is evident in their fire management practices, which play a crucial role in promoting socioecological balance. By engaging in local fire management, these communities actively contribute to the preservation of local ecosystems, biological diversity, and harmony between society and the natural environment. However, many of these studies overlook the assessment of burn severity levels that local fire management can produce, resulting in insufficient attention to the effects of fire on natural resources [29]. In this context, Ecuador faces a scarcity of information on the severity of wildfires due to ancestral fire management, with only one available study involving the Saraguro indigenous community [30]. This lack of information hinders the confirmation of the impacts of such fires, both positive and negative. The significance of this issue lies in the fact that globally, several studies indicate that low-severity fires benefit soil biogeochemical cycles by increasing organic matter and essential nutrients such as phosphorus and nitrogen, while high-severity fires can be lethal. However, some reports highlight damages associated with the frequency of fires, even when they are of low severity, as detrimental [31]. Therefore, further research on this topic is needed to clarify the impacts of fires produced by human fire management on Ecuador's natural resources.

Ecuador, a multiethnic and multicultural country, has a history marked by migratory and mestizo processes involving whites, mestizos, native Amerindians, and Afro-Ecuadorians [32]. Both mestizos and native Amerindians utilize traditional fire management in their agricultural and forest management activities, as demonstrated by recent research [29, 33]. For example, Díaz et al. [30] highlighted the cultural significance and ecological effects of the human use of fire by the Saraguro indigenous people in the páramo ecosystem of southern Ecuador. Therefore, it is of paramount importance to undertake research programs in Ecuador aimed at integrated fire management, considering aspects such as traditional fire use, severity levels, burning frequency, and their effects on nature and human well-being. This endeavor becomes crucial given the diversity of ecosystems and ethnicities in Ecuador, spread across four natural regions encompassing the Coast, the Sierra, the Amazon, and the Galápagos Islands [23].

On the other hand, in addition to understanding the ethnobiological use of fire, it is crucial to conduct monitoring of the impacts of wildfires using widely recognized tools such as remote sensing, which enjoys global recognition [34]. In many countries, this technique characterizes wildfire regimes, evaluates meteorological conditions, and determines severity levels [35, 36]. However, in Ecuador, there is a notable absence of comprehensive studies in this field. Cabrera et al. [37] and Reyes and Loján [38] proposed reproducible remote sensing methodologies for the semi-automatic identification of wildfires. Carrión-Paladines et al. [39] correlated remote sensing with soil properties in high-Andean shrublands. Yangua-Solano et al. [40] have explored the impact of low severity on pioneer species, while Díaz et al. [30] have linked remote sensing to the use of fire by the Saraguro indigenous community. Despite these advancements, Ecuador lacks additional studies that comprehensively address the use of this methodology alongside the effects of wildfires. Additionally, it faces a knowledge gap regarding wildfires and their effects, due to the diversity of ecosystems with unknown susceptibility or adaptation to fire [30, 41]. The limited presence of meteorological stations hinders research in this area [42], posing a significant challenge [43]. Meteorological data from NASA's POWER reanalysis compensate for this limitation, proving reliable at regional and national scales [36, 44]. By using satellite-collected information, these models enhance wildfire research and contribute to a better understanding of climatic phenomena, as evidenced by studies like Kanga et al. [45] and Carrión-Paladines et al. [39] on wildfires in Himachal Pradesh and high-Andean shrublands, respectively. These examples underscore the importance of these methods in advancing wildfire research and improving our understanding of climatic phenomena in various geographical areas.

The aim of this study was to examine potential differences and similarities in human fire use across the Coast, Sierra, and Amazon regions of Ecuador. Based on this aim, we hypothesized that significant variations exist in fire use practices among these regions, stemming from differences in ecosystems, agricultural practices, and cultural and socioeconomic needs of each region. To achieve this goal, semi-structured interviews were conducted to gather local knowledge, optimal weather conditions for burning were identified, and fire severity was assessed. Additionally, an analysis of current regulations was conducted to identify potential environmental infringements in the study areas and recommend reforms tailored to the specific realities of each region's inhabitants. Finally, local knowledge was integrated to determine its alignment with scientific approaches. These findings of this research are vital for decision-makers as they enhance understanding of human fire use and facilitate the development of more effective, inclusive, and sustainable integrated fire management programs for Ecuador's diverse ecosystems.

## Materials and methods

### Study area on the continental Ecuador

**Fig. 1:**
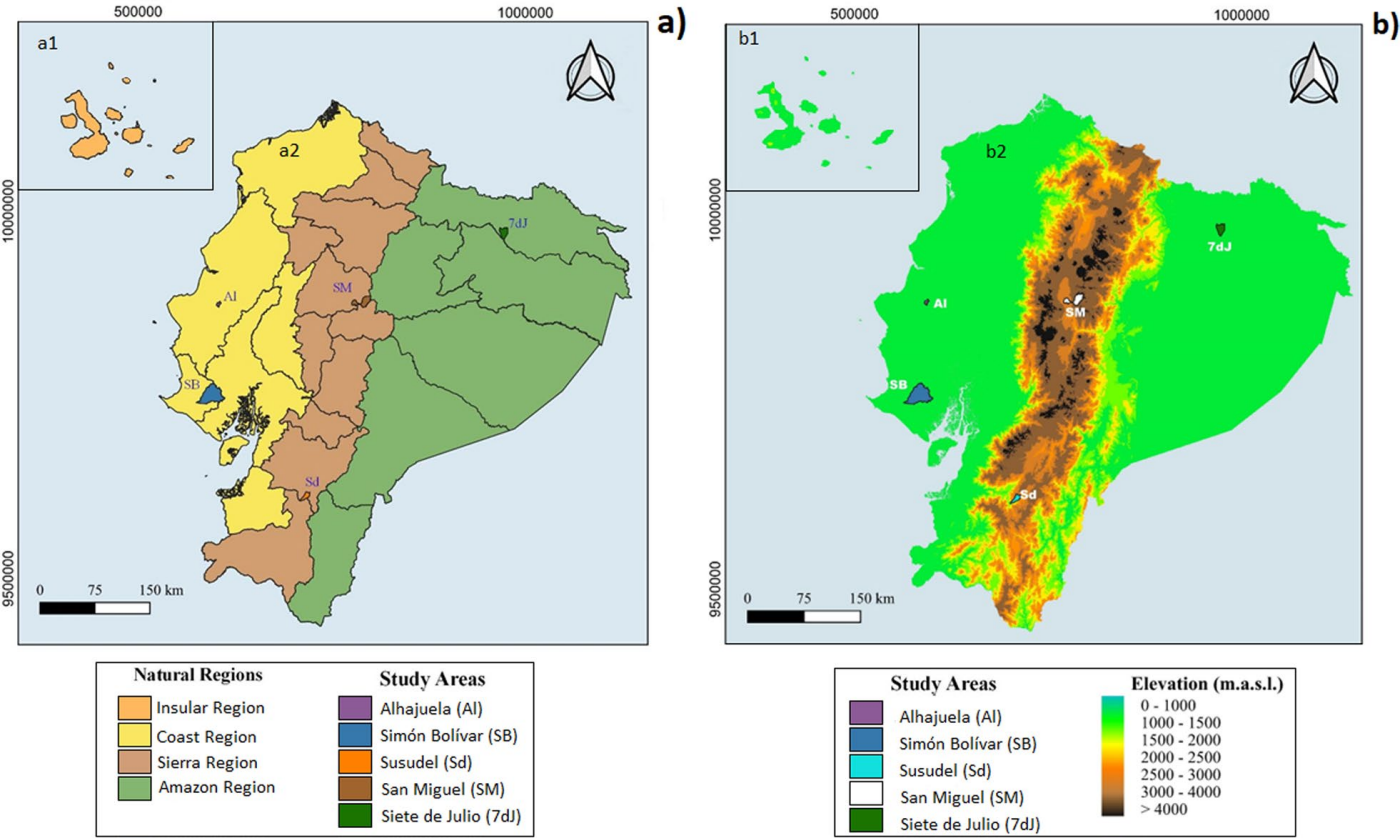
Location of the study areas. **a** Map of continental Ecuador showing: **a1** the insular region or Galapagos islands; **a2** the three natural regions where the research was carried out, together with their respective codes correspond to the coast region, the Sierra region, and the Amazon region. In addition, the names of the localities where the studies were carried out are indicated: Alhajuela (Al), Simón Bolívar (SB), Susudel (Sd), San Miguel (SM), and Siete de Julio (7dJ). **b** Digital elevation model (DEM) of continental Ecuador

The study covered three natural regions of Ecuador, excluding the Galapagos Islands due to their protected area status. The other natural regions such as the Coast, Sierra, and Amazon are each characterized by their unique geography, climate, and ecosystems [46]. From the Coast and Sierra regions, two parishes each were selected because these areas are where most wildfires occur [46, 47]. From the Amazon region, one parish was selected because this area has high humidity, which resulting in a low incidence of wildfires [46]. All the parishes studied were chosen based on geographic, climatic, and accessibility criteria (Fig. 1, Table 1).

**Table 1:** Main characteristics of the parishes studied in Ecuador's three natural regions

Natural region	Province	Parish	Code	Extension (km^2^)	Ubication		Meteorology		Ecological formation	References
					Universal Transverse Mercator (UTM)		Temp	Rainfall		
					x	y	°C annual average	mm/year		
Coast	Manabí	Alhajuela	Al	23,2	0579996.00	9,883,520.00	24	500–1000	Tropical very dry forest, and tropical dry forest	Holdridge [48]; GAD of Alhajuela [49]
	Santa Elena	Simón Bolívar	SB	557,5	571,286.55	9,758,607.50	25,2	800–1200	Tropical thorny forest, tropical very dry forest, and tropical dry forest	GAD of Simón Bolívar [50]; Holdridge [48]
Sierra_Andes	Azuay	Susudel	Sd	72,42	701,956.00	9,623,550.00	20,0	250–500	Semi-deciduous shrubland of the South Valleys	Aguirre et al. [51]; GAD Parroquial of Susudel [52]
	Cotopaxi	San Miguel	SM	225,0	768,137.74	9,884,341.11	13,0	500–1000	Montane Rainforest	Hernández Reinoso [53]
Amazon	Sucumbíos	7 de julio	7dJ	123,6	301,373.80	9,979,449.80	27,0	2000–4000	Tropical rain forest	Izquierdo et al. [47]; Cañadas Cruz, [54]

### Specific study areas

**Fig. 2:**
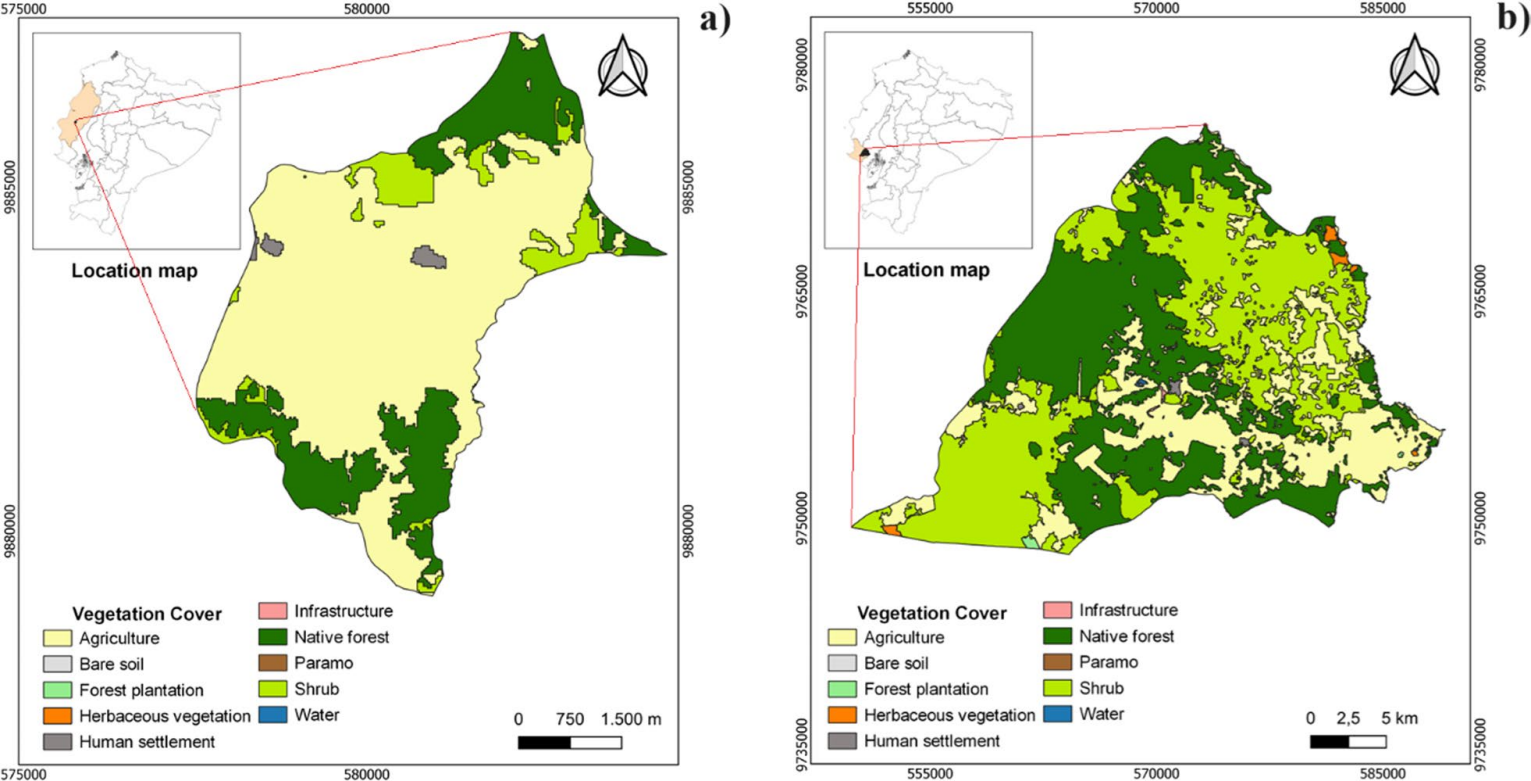
Vegetation cover. **a** Alhajuela Parish (Al); and **b** Simón Bolívar Parish (SB). The parishes belong to the coast region of continental Ecuador

In the coast region, two parishes were selected: Alhajuela and Simón Bolívar (Al and SB, respectively) (Fig. 2; Table 1). Al is in the province of Manabí, in the Portoviejo canton, with an average temperature of 24 °C and an annual precipitation range of 500–1000 mm. The parish features very dry tropical forests and tropical dry forests ecologically, making it a vital area for diverse agricultural activities, including corn (*Zea mays*), peanuts (*Arachis hypogaea*), cassava (*Manihot esculenta*), cocoa (*Theobroma cacao*), plantain (*Musa paradisíaca*), vegetables and legumes [48, 49] (Table 1).

On the other hand, SB belongs to the province of Santa Elena and has an average temperature of 25.2 °C, with an annual precipitation range of 800–1200 mm [50]. This parish is home to tropical thorn forests, very dry tropical forests, and tropical dry forests [48], with the main economic activities being agriculture and cattle ranching (*Bos taurus*). The main crops in the area are tomato (*Solanum lycopersicum*), cassava (*Manihot esculenta*), papaya (*Carica papaya*), watermelon (*Citrullus lanatus*), corn (*Zea mays*), and fruit trees such as plum (*Spondias purpurea*) [50].

**Fig. 3:**
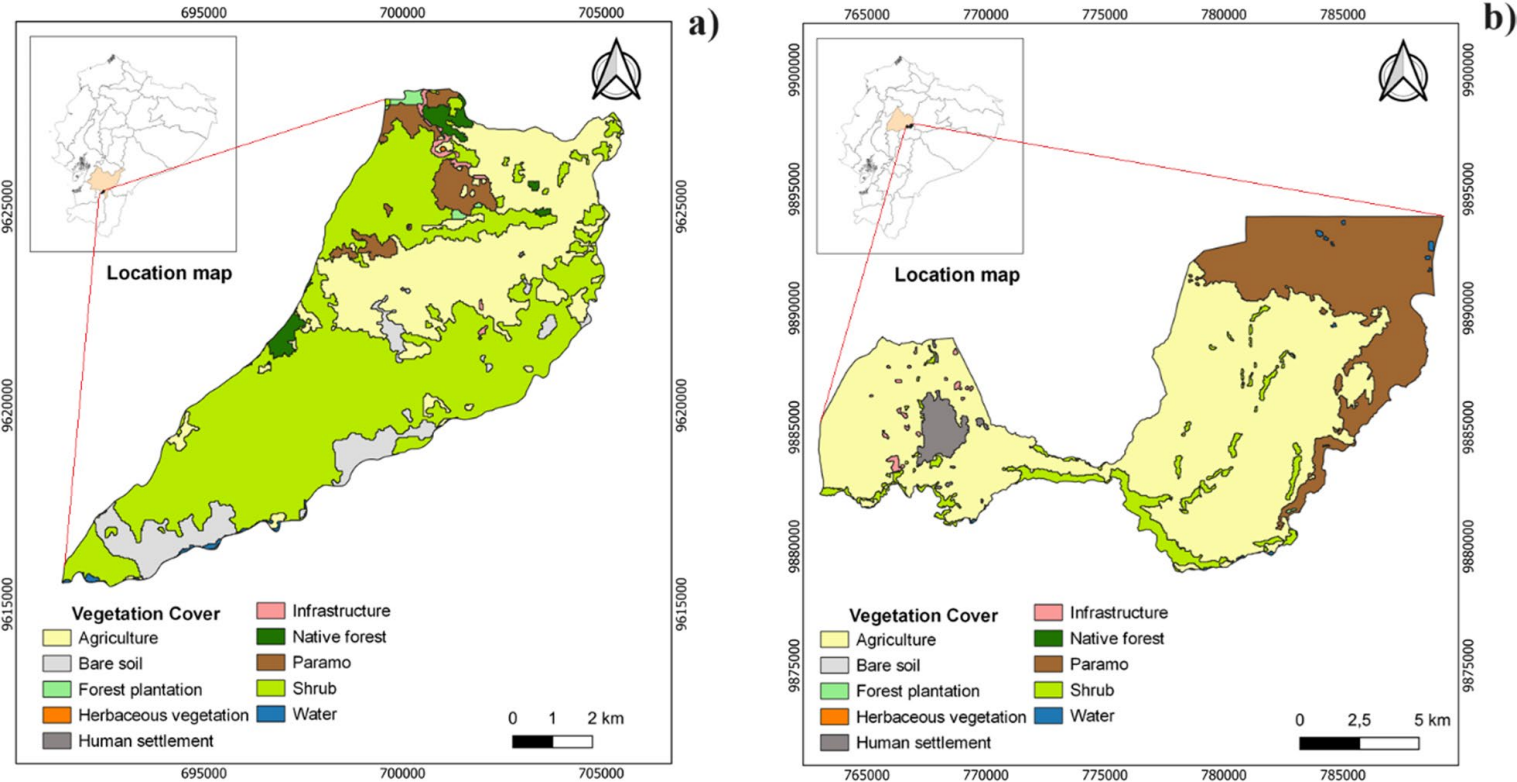
Vegetation cover. **a** Susudel Parish (Sd) and **b** San Miguel Parish (SM). The parishes belong to the Sierra region of continental Ecuador

The parishes of Susudel and San Miguel (Sd and SM, respectively) are in the Sierra region (Fig. 3, Table 1). Sd is part of the Azuay province and has an average temperature of 20 °C, with an annual precipitation range ranging from 250 to 500 mm [51, 52]. In this parish, you can find the semi-deciduous shrublands of the Southern Valleys [51], where the main economic activities include livestock farming and the cultivation of perennial plants such as tree tomato (*Solanum betaceum*), as well as short-cycle crops like corn (*Zea mays*) and potatoes (*Solanum tuberosum*). In the Sierra region, there is also SM, which is part of the Cotopaxi province and has an average temperature of 13 °C, with an annual precipitation range between 500 and 1000 mm [53]. In this parish, you can find the ecological formation of Montane Rainforest. The main economic activities are livestock farming and the cultivation of short-cycle crops like corn, potatoes, and lupine (*Lupinus mutabilis*).

**Fig. 4:**
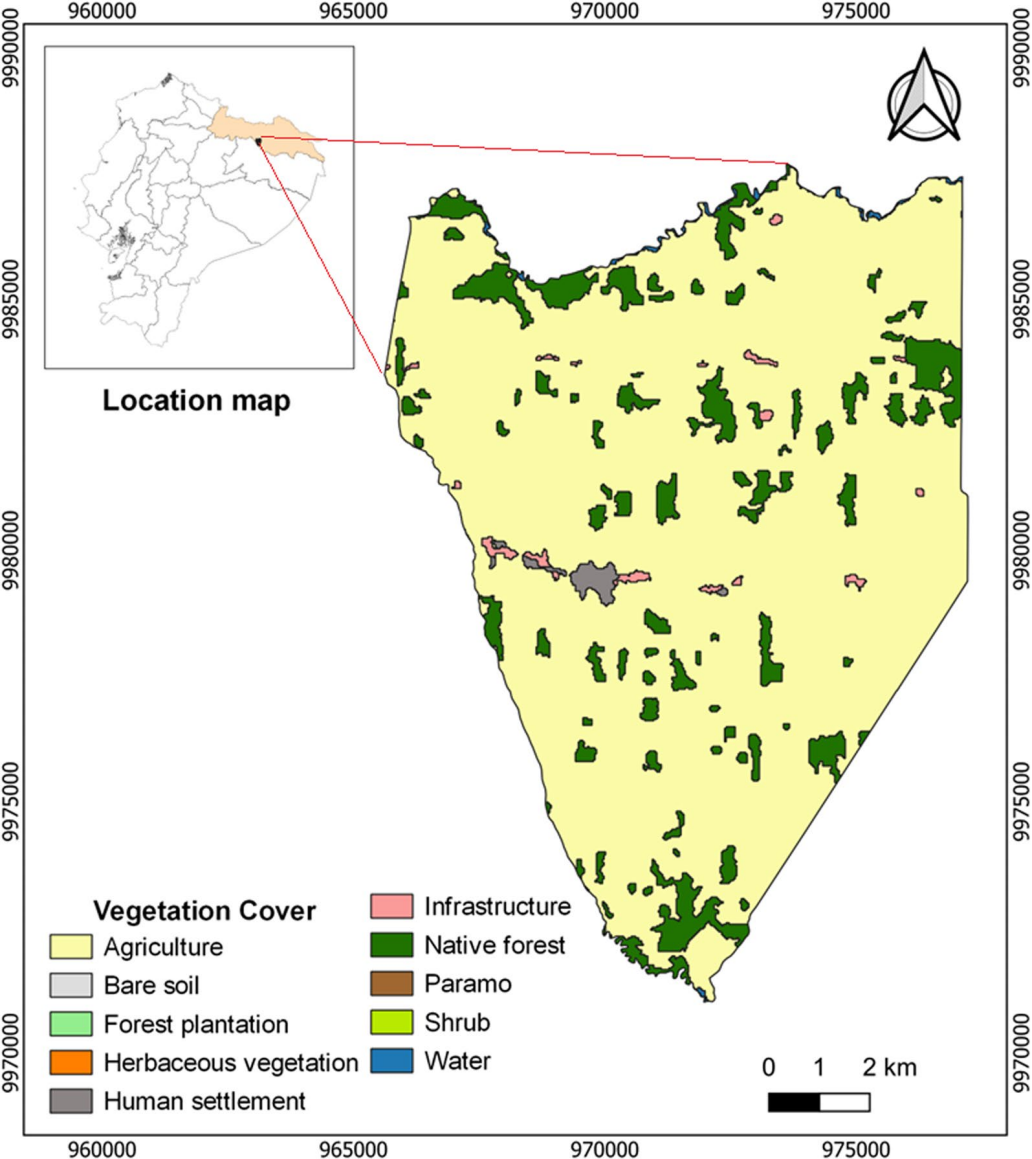
Vegetation cover of the 7 de Julio Parish (7dJ). The parish belongs to the Amazon region of continental Ecuador

Finally, in the Amazon region, parish 7dJ is in the Sucumbíos province and is characterized by having an average annual temperature of 27 °C, with an annual precipitation ranging from 2000 to 4000 mm [47, 54]. This parish is known for its tropical rainforest environment, where various economic activities take place, including oil exploitation, livestock farming, and agriculture, which includes the cultivation of coffee (*Coffea arabica*), cocoa (*Theobroma cacao*), African palm (*Elaeis guineensis*), and corn [55] (Fig. 4, Table 1).

### Collecting data on human use of fire in three natural regions of Ecuador

The research on the human use of fire was conducted with the collaboration of parish councils, given their fundamental role in the management and development of local communities in Ecuador [30]. In collaboration with the president of each board and his team, briefing meetings were organized to present the research objectives and the content of the semi-structured survey containing open and closed questions (see the Additional File 1_ Appendix I for details). Subsequently, a local liaison, designated by each parish council, facilitated the identification of survey application areas and encouraged residents to participate in data collection, fostering trust and knowledge sharing. The survey targeted people aged 25 to 75 years affiliated with each parish council, covering both men and women [56]. In total, 791 collaborators from the parishes studied, predominantly of mixed race, were surveyed (Table 2). For the selection of the number of survey collaborators, the standard formula was used to calculate the sample size (simple random sampling) based on the population of each parish, as has been done in recent research [57].

**Table 2:** Number of individuals surveyed according to their ethnic identity to evaluate the human use of fire in three natural regions of Ecuador

Natural region	Parish	Code	Number of people surveyed			Ethnic identity (%)			
			Women	Men	Total	Mixed-race	Indigenous	Other	Total
Coast	Alhajuela	Al	50	58	108	100	0	0	100
	Simón Bolívar	SB	81	85	166	100	0	0	100
Sierra_Andes	Susudel	Sd	56	78	134	100	0	0	100
	San Miguel	SM	123	77	200	47.5	52.5	0	100
Amazon	7 de julio	7dJ	86	97	183	97.8	2.2	0	100
Total			396	395	791				

Data from individuals, both women and men, aged between 25 and 75 years were considered

The survey covered various aspects, such as farmers' social profiles, primary agricultural practices, methods of fire, ancestral burning knowledge, ignition patterns, perceptions of fire impacts on natural resources, preferred times of use, and levels of training received. Based on this information and using the participant observation method, community fire calendars were developed, in line with the model of McKemey et al. [58]. Subsequently, these calendars were crossed with meteorological data, following the approach of Díaz et al. [30] which resulted in the formulation of area-specific fire calendars or each area. This analysis allowed the identification of patterns and relationships between local practices and environmental conditions.

To validate the developed fire calendar, discussions were held with farmers and members of each parish council, as has been done in recent research [30, 58]. These discussions ensured the relevance and accuracy of the calendar in relation to traditional practices and environmental conditions specific to each parish. Adjustments or improvements were made based on comments received from stakeholders. This comprehensive methodology facilitated the creation of a practical and culturally relevant tool to guide fire management practices in the different study regions in Ecuador.

### Quantitative data analysis

The historical uses and impacts of fire were assessed by computing two metrics: the level of information fidelity (LIF) and the informant's consensus factor (ICF). The LIF was determined using a formula sourced from previous studies [30, 59]:

$${\text{LIF}} = \left( {\frac{{{\text{Ip}}}}{{{\text{Iu}}}}} \right)*100$$

where Ip represents the count of informants indicating a specific characteristic related to fire and Iu represents the count of informants indicating all characteristics related to fire use. A higher LIF value justifies the frequent use of a specific fire-related characteristic, indicating its prevalence. On the contrary, a lower LIF value means a lower frequency of the characteristic in the use of fire. In essence, LIF serves as a quantitative metric, reflecting the degree of preference for a particular characteristic of fire use compared to others.

The ICF was employed to quantify the consensus or agreement among all informants (respondents) regarding each fire use characteristic. The ICF in this study was computed using the formula outlined by Khan et al. [60] and Díaz et al. [30]:

$$\text{IFC}=\left(\text{Nt}-\text{Nur}\right)/(\text{Nt}-1)$$

where Nt represents the total number of informants surveyed and Nur represents the subset of informants who indicate a specific or main use of fire. The ICF yields results within the range of 0 to 1. The lowest values, close to 0, indicate a similarity in the characteristics of fire use, which means exchange of information or consensus between informants. Conversely, higher values close to 1 suggest randomness in the execution of a fire use characteristic or a lack of information sharing, indicating a lack of consensus among informants regarding its use.

### Legal context of wildfires in Ecuador

Due to the prevailing legal gaps in Ecuador regarding the inadequate classification of wildfire severity and the absence of acknowledgment of ecosystems' fire adaptability, we conducted an in-depth analysis of existing legislation on penalties. Our review spanned all legal aspects, from the national Constitution to Municipal Ordinances, utilizing the Vlex search engine (https://app.vlex.com/), as per recent research practices [61]. Additionally, we compared Ecuador's legislation with that of South American countries and other pertinent regions, following Díaz et al. [30] methodology.

### Determination of the fire season

The fire season was established using NASA meteorological data, accessed at https://power.larc.nasa.gov/data-access-viewer/ (accessed October 2023). Analyzed meteorological variables included temperature (°C), precipitation (mm), relative humidity (%), and wind speed (km/h) [39, 62]. Parish-specific geographical coordinates, collected during surveys on local fire usage patterns, guided weather data retrieval. Subsequently, historical climographs for each parish were generated from a 41-year dataset (1981–2021). Monthly averages of precipitation, relative humidity, temperature, and wind speed were computed, identifying two climatic phases: the rainy period and the annual drought period [63]. Historical and annual climographs were employed to interpret traditional fire usage and assess fire severity indices.

### Determination of severity of wildfires

The severity of wildfires was calculated using images from the Sentinel 2B satellite with its multispectral MSI sensor [64]. The years 2019, 2020, 2021, and 2022 were considered, selecting dates with lower cloud cover (< 20% cloudiness) during the last months of the year, when the highest incidence of wildfires occurs [65]. Additionally, the Normalized Burn Ratio (NBR) was employed, specifically designed to identify areas affected by fires through their spectral signature [66]. For this analysis, the following formula was applied [67].

$$\mathbf{N}\mathbf{B}\mathbf{R}= \frac{({R}_{\text{NIR}}-{R}_{\text{SWIR}})}{({R}_{\text{NIR}}+{R}_{\text{SWIR}})}$$

$${{\varvec{R}}}_{\mathbf{N}\mathbf{I}\mathbf{R}}=\text{reflectivity}\, \text{in} \,\text{the} \,\text{band} \,\text{NIR} \left(B8A\right)$$

$${{\varvec{R}}}_{\mathbf{S}\mathbf{W}\mathbf{I}\mathbf{R}}=\text{reflectivity} \,\text{in} \,\text{the}\, \text{band} \,\text{SWIR} (B12)$$

Likewise, dNBR, the difference between pre-fire and post-fire (NBR Pre-fire–NBR Post-fire) was calculated to estimate the severity using the respective formula [35].

$$\mathbf{d}\mathbf{N}\mathbf{B}\mathbf{R}= ({\text{NBR}}_{1}-{\text{NBR}}_{2})$$

$${\mathbf{N}\mathbf{B}\mathbf{R}}_{1}=\text{pre}-\text{fire} \text{burned} \text{area} \text{index}$$

$${{\varvec{N}}{\varvec{B}}{\varvec{R}}}_{2}=\text{post}-\text{fire} \text{burned} \text{area} \text{index}$$

## Results

### Traditional use of fire

In Table 3, the findings of this study are summarized, highlighting differences and similarities in fire use across Ecuador's natural regions. For instance, in coast areas, agricultural activities are the main cause of wildfires, accounting for 80% in Al and 58.4% in SM. In contrast, poorly extinguished campfires prevail during camping in forests in the Sierra and the Amazon, contributing 88.1% and 96.7% of fires in Sd and 7dJ, respectively.

**Table 3:** Outstanding characteristics of the respondents regarding the use of fire in three natural regions of Ecuador

Characteristics/use of fire		Coast Region				Sierra Region				Amazon Region	
		Alhajuela		Simón Bolívar		Susudel		San Miguel		7 de Julio	
		LIF (%)	ICF	LIF (%)	ICF	LIF (%)	ICF	LIF (%)	ICF	LIF (%)	ICF
Reasons for the wildfires in the area	Burns due to agricultural activities	80	0.2	27.7	0.8	7.2	0.9	1	1	0	1
	Natural causes	0	1	13.9	0.9	2.2	1.0	46.2	0.5	1.1	1
	Burning of household garbage	20	0.9	58.4	0.5	2.5	1.0	23.2	0.9	2.2	1
	Campfires	0	1	0	1	88.1	0.2	29.6	0.9	96.7	0
	*Percentage*	100		100		100		100		100	
Land preparation for agricultural activities	Vegetation clearing and burning	24.1	0.8	73.5	0.3	5.9	0.9	15.6	0.6	33.9	0.7
	Fallow land	0.9	1	0	1	18.3	0.7	0.6	1	0	1
	Mechanized tillage	44.4	0.6	4.8	1	33.8	0.5	36.7	0.1	19.3	0.8
	Manual tillage	15.7	0.9	2.4	1	20	0.7	35.5	0.1	44.9	0.6
	Tillage with oxen	0	1	0	1	18.3	0.7	8.7	0.8	0.0	1
	Traditional burning	8.2	0.8	9.3	0.8	1.9	0.9	1.8	1	1.0	1
	Agricultural burning (burning crop residues)	7,0	1	10.0	0.8	1.8	0.9	1.0	1	1.0	1
	*Percentage*	100		100		100		100		100	
Frequency of burning in the area	Every month	0	1	52.4	0.5	2.5	1	0.7	1	1.1	1
	Every three months	0	1	24.7	0.8	2.5	1	21.1	0.7	7.7	0.9
	Every six months	11	0.9	19.3	0.8	2.5	1	38.2	0.5	20.9	0.8
	Each year	89	0.2	3.6	1	92.5	0	40	0.4	70.3	0.3
	*Percentage*	100		100		100		100		100	
Purpose of fire use	To improve soil fertility	30.6	0.7	29.6	0.7	8.9	0.9	16.1	0.7	24.2	0.8
	By family tradition	5.6	1	17.6	0.9	5.2	1	0.3	1.0	3.3	1.0
	To improve crop yields	5.6	1	25.3	0.8	13.3	0.8	30.0	0.5	5.5	1.0
	For stubble removal	58.3	0.4	25.3	0.8	69.6	0.2	53.6	0.6	67.0	0.3
	For eliminating pests	0	1	2.2	1	3	1	0	1	0	1
	*Percentage*	100		100		100		100		100	
Contribution of ash to the soil	Provides nutrients and minerals	0	1	45.1	1	23.6	0.2	0	1	73.6	0.3
	Used as fertilizer	55.6	0.4	24.2	1	5	1	0	1	0	1
	Improves crop quality	2.8	1	16.8	1	32.1	0.3	100	0.0	12.1	0.9
	Eliminates pests	0	1	13.9	1	34.3	0.3	0	1	14.3	0.9
	Ignorance of use	41.7	0.6	0	1	5	1	0	1	0	1
	*Percentage*	100		100		100		100		100	
Instruments used to light the fire	Matches	65.7	0.3	100	0	94.9	0.2	99.5	0	76.9	0.2
	Handcrafted lighters	34.3	0.7	0	1	4.4	0.9	0.5	1	7.2	0.2
	Firefighter torches	0	1	0	1	0.7	1.0	0	1	0	1
	Handmade torches	0	1	0	1	0.0	1.0	0	1	15.9	0.9
	Flares	0	1	0	1	0.0	1.0	0	1	0	1
	*Percentage*	100		100		100		100		100	
Ignition techniques for fire starting	Stacks the dry material to be burned	96.3	0	75.9	0.2	59.9	0.4	100	0	65.9	0.3
	Light the fire in the form of a girdle	2.8	1	24.1	0.8	9.7	0.9	0	1	4.4	1
	Light the fire in the form of dots	0.9	1	0	1	30.4	0.7	0	1	29.7	0.7
	*Percentage*	100		100		100		100		100	
Patterns for fire lighting	Burning on flat terrain	35.2	0.7	83.1	0.2	55.6	0.4	68.8	0.4	56	0.4
	Burning on sloping terrain	45.4	0.6	4.8	1	23.4	0.7	30.9	0.6	2.2	1
	Start burning from the top down	0	0	7.2	0.9	3.7	1.0	0	1	7.7	0.9
	Starting to burn from bottom to top	19.4	0.8	4.8	1	17.5	0.8	0.3	1	34.1	0.7
	*Percentage*	100		100		100		100		100	
Control measures to extinguish the fire	Clearing of vegetation around the site	33.3	0.7	41.9	0.4	34.0	0.6	1.9	1.0	12.1	0.9
	Call the fire department	9.3	0.9	0.4	1	1.3	1	12.1	1	17.6	0.8
	Ask the community for help	36.1	0.6	35.5	0.4	6	0.9	15.3	0.7	20.9	0.8
	Extinguish flames with fire-resistant branches dampened with water	16.7	0.8	17.5	0.8	42.0	0.5	48.5	0.1	17.6	0.8
	Trample the burned vegetation and trample the sparks	0	0	0.9	1	8.0	0.9	21.2	1	3.3	1.0
	Builds a trench to mineral soil around the site	4.6	1	3.9	1	8.3	0.9	1.1	1	28.6	0.7
	*Percentage*	100		100		100		100		100	
Useful aspects for fire lighting	Considers wind speed and direction	87	0.1	91	0.1	22.3	0.8	10.9	0.8	39.6	0.6
	Burns when there is enough sun	0	1	0.6	1	8.3	1.0	34.2	0.4	8.5	0.6
	It burns when it has not rained	4.6	1	4.8	1	32.3	0.8	28.9	0.5	1.8	0.7
	Burn when soil and vegetation is dry	8.3	0.9	3.6	1	36.7	0.7	26	0.5	50.3	0.3
	*Percentage*	100		100		100		100		100	
Optimum months for the use of fire for agricultural burning	January–February	0	1	0	1	1.2	0.9	2.4	0.9	19.8	0.8
	March–April	0	1	7.8	0.9	4.5	0.9	25.5	0.7	4.4	1
	May–June	0	1	0	1	24	0.7	26	0.7	7.7	0.9
	July–August	0	1	23.5	0,0.8	34.6	0.7	24.6	0.7	3.3	1
	September–October	74.1	0.3	65.1	0.4	24.6	0.7	9.6	0.9	54.2	0.5
	November–December	25.9	0.7	3.6	1	10.6	0,0.8	11.6	0.9	11	0.9
	*Percentage*	100		100		100		100		100	

The responses correspond to direct users of ecosystems in rural parishes in the coast, Sierra, and Amazon regions of Ecuador. Fire uses were evaluated by computing two metrics: the level of information fidelity (LIF) and the informant consensus factor (ICF)

Land preparation practices leading to wildfires also vary regionally. In coast regions, deforestation and vegetation burning are prominent (Al: 24.1%; SB: 73.5%), followed by traditional burning (Al: 8.2%; SB: 9.3%), and agricultural burning, including crop residues (Al: 7.0%; SB: 10.0%). In the Sierra, deforestation and vegetation burning have low percentages (Sd: 5.9%; SM: 15.6%), while in the Amazon, they account for 33.9%.

Fire frequency also varies. Annual fires prevail in Al (89%), while SB reports monthly (52.4%) and quarterly (24.5%) frequencies. In the Sierra, Sd has the highest annual frequency (92.5%), while SM and the Amazon experience fires every six months (38.2% and 20.9%, respectively).

A key aspect is the purpose of fire use, similar across the three regions. The main goal is to clear crop stubble (Al: 58.3%; SB: 25.3%; Sd: 69.6%; SM: 53.6%; 7dJ: 67.0%) and improve soil fertility (Al: 30.6%; SB: 29.6%; 7dJ: 24.2%). Ashes serve as a nutrient source (SB: 45.1%; Sd: 23.6%; 7dJ: 73.6%) and fertilizer (Al: 56.6%; SB: 24.2%). Common practices include using matches, piling dry material, and burning on flat ground.

Furthermore, respondents consider factors like wind speed and direction during burns. However, the construction of ditches around the land as a fire control measure is absent in all three regions. These results underscore the need for region-specific fire management strategies tailored to the diverse practices and challenges observed in Ecuador.

### Legal regulations

Table 4 provides an overview of the existing legal regulations governing wildfire prevention in the examined districts. It is noteworthy that none of these regulations, ranging from constitutional provisions to municipal ordinances directly applicable in the parishes, incorporate crucial ecological concepts necessary for a comprehensive understanding of the wildfire issue in each area. In all the studied parishes, regulations primarily focus on punitive measures as stipulated in Article 246 of the COIP—Integral Penal Code [68]. This code considers the negligent initiation of uncontrolled burning leading to wildfires as a punishable offense, with imprisonment ranging from 3 to 6 months and, in cases of fatalities, 13 to 16 years. However, the parishes only ensure the timely provision of prevention and firefighting services in designated wildfire defense zones (Table 4). Municipal ordinances adopting an integrated fire management (IFM) approach are lacking, in regulating the implementation of controlled burning plans and, in specific contexts, prescribed burning plans considering climatic factors for specific fire seasons, severity levels, and crucial ancestral knowledge used in traditional burning (Table 3). It is noteworthy that only the Al parish refers to prevention systems, including fuel volume control, controlled burns, and the creation of firebreaks. Both the municipality and the parish commit to researching sustainability, resilience, and carbon sequestration, as well as intervening and restoring degraded areas by introducing native species. Consequently, Ecuador lacks a legal framework that promotes the establishment of an improved Municipal IFM system.

**Table 4:** Current regulatory framework for wildfire control. The regulations applicable to each parish under study are listed, along with detailed explanations and corresponding legal references

Natural region of Ecuador	Parish	Municipal ordinance	Current	Brief explanation	Concordances
Coast	Alhajuela	1. ORDINANCE AMENDING THE MUNICIPAL CODE BOOK 5 ENVIRONMENTAL COMPONENT, WHICH INCORPORATES THE UNNUMBERED TITLE “OF THE PROTECTION, CONSERVATION, USE AND MANAGEMENT OF URBAN TREES AND INFRASTRUCTURE IN THE CANTON OF PORTOVIEJO”	Official Gazette—Special Edition N° 861—Monday, May 8, 2023	This ordinance refers to urban infrastructure and the planning of urban trees. Concerning to fires, it limits itself to mentioning on which part of the urban furniture the structural firefighting equipment should be placed, as well as the emergency actions to be taken by administrative and operative management in parks with forests or forest plantations (wildfires)	* Constitution of the Republic of Ecuador
					* Organic Code of Territorial Organization, Autonomy and Decentralization
					* Organic Code of the Environment
					* Ministerial Agreement 018 of February 23, 2016, of the Ministry of Environment
					* Ministerial Agreement 059 of March 23, 2017
					* Ordinance Regulating the Institutional Development of the Canton of Portoviejo
		2. ORDINANCE INCORPORATING THE PORTOVIEJO 2035 PLAN INTO MUNICIPAL REGULATIONS	Official Gazette—Special Edition No. 1611—Friday, July 9, 2021	It regulates the protection of commercial land, homes, and businesses against fires. Regarding wildfires, it refers to the generation of prevention systems, that is: fuel volume control, controlled burns, and firebreak strips, it will also oversee the research of sustainability, resilience, and carbon sequestration processes, as well as the intervention and recovery of degraded areas with native species	* Constitution of the Republic of Ecuador
					* Organic Code of Territorial Organization, Autonomy and Decentralization
					* Organic Law of Land Management, Land Use and Management
					* Organic Planning and Public Finance Code
		3. ORDINANCE AMENDING THE ORDINANCE REGULATING THE FEES FOR SERVICES RENDERED BY THE PORTOVIEJO FIRE DEPARTMENT “CORONEL JOSÉ ANTONIO MARÍA GARCÍA PINOARGOTE”	Official Gazette—Third Supplement No. 400—Monday, March 1, 2021	Establishes requirements and fees for operating permits, drawing of plans and other services provided by the Fire Department, so that establishments comply with structural fire safety standards. It does not refer to wildfires	* Constitution of the Republic of Ecuador
					* Organic Code of Territorial Organization, Autonomy and Decentralization
					* Fire Defense Law
					* General Regulations of the Fire Defense Law
					* Resolution No. 0010-CNC-2014 COMPETENCY FIRE SERVICE FOR THE BENEFIT OF DECENTRALIZED GOVERNMENTS
	Simón Bolívar	1. SUBSTITUTE ORDINANCE OF INTEGRATION INTO THE MUNICIPAL GOVERNMENT AND OPERATION OF THE FIRE DEPARTMENT OF THE SANTA ELENA CANTON	Official Gazette No. 523—May 27, 2015	It regulates the inspection and granting of permits for constructions and buildings as well as compliance with fire prevention and/or closure regulations. With respect to wildfires, this law states that it fights wildfires and will guarantee the timely provision of its services in areas of defense against wildfires	* Constitution of the Republic of Ecuador@* Fire Defense Law@* Organic Code of Territorial Organization, Autonomy and Decentralization
Sierra	Susudel	1. SUBSTITUTE ORDINANCE, WHICH REPEALS THE ORDINANCE OF ASSIGNMENT OF THE FIRE DEPARTMENT OF THE CANTON OF SAN FELIPE DE OÑA	Official Gazette—Special Edition No. 850—Friday, April 28, 2023	The Fire Department should carry out structural and wildfire prevention campaigns. In addition to fighting wildfires	* Constitution of the Republic of Ecuador
					* Organic Code of Territorial Organization, Autonomy and Decentralization
					* Organic Code of the Public Security and Public Order Entities
					* Fire Defense Law
	San Miguel	1. At the parish and canton levels, there are no regulations that refer to wildfires, but only to forest land located in rural areas	Official Gazette—Special Edition Nº 1916—Friday, January 28, 2022	It deals with the determination of the additional tax that finances the firefighting service for the benefit of the Fire Department of Canton Salcedo	* Constitution of the Republic of Ecuador
					* Organic Code of Territorial Organization, Autonomy and Decentralization
					* Tax Code
					* Organic Law on Rural Lands and Ancestral Territories
Amazon	7 de Julio	1. ORDINANCE FOR THE CREATION AND DELIMITATION OF THE AREA OF CONSERVATION AND SUSTAINABLE USE OF THE PANTHER FOREST (ACUSBLP)	Official Gazette—Special Edition Nº 89,843—Friday, June 2, 2023	This ordinance indicates that when wildfires that affect natural vegetation cover are caused, they will be sanctioned in accordance with the provisions of the Integral Penal Code	* Constitution of the Republic of Ecuador
					* Organic Integral Penal Code
					* Organic Code of Territorial Organization, Autonomy and Decentralization
					* Organic Code of the Environment
		2. ORDENANZA QUE REGULA LA GESTIÓN DE LOS SERVICIOS DE PREVENCIÓN, PROTECCIÓN, SOCORRO Y EXTINCIÓN DE INCENDIOS EN EL CANTÓN SHUSHUFINDI, DEL CUERPO DE BOMBEROS ADSCRITO AL GAD MUNICIPAL DE SHUSHUFINDI	Official Gazette—Special Edition No. 243—March 17, 2016	It is indicated that among the duties and attributions of the Shushufindi Fire Department is that of fighting wildfires	* Constitution of the Republic of Ecuador
					* Organic Code of Territorial Organization, Autonomy and Decentralization
					* Fire Defense Law
					* General Regulations of the Fire Defense Law
					* Ordinances issued by the Autonomous Decentralized Municipal Government of Canton Shushufindi

### Meteorological influences on wildfire season 

**Fig. 5:**
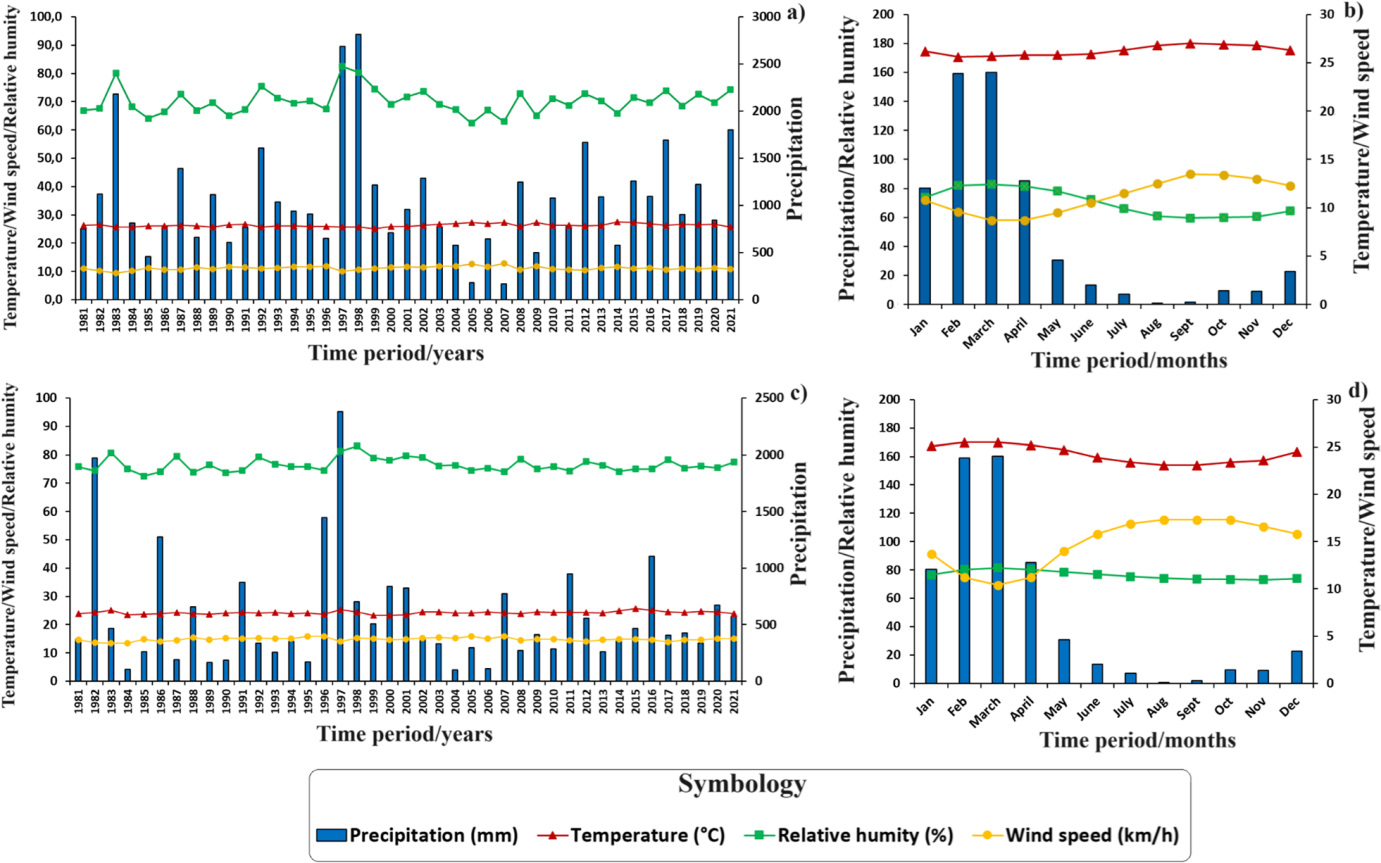
Climographs of the Ecuadorian coast, generated with NASA meteorological data (period: 1981–2021). **a** Historical climograph of Al; **b** Climograph with monthly values of Al; **c** Historical climograph of SB; and **d** Climograph with monthly values of SB

**Fig. 6:**
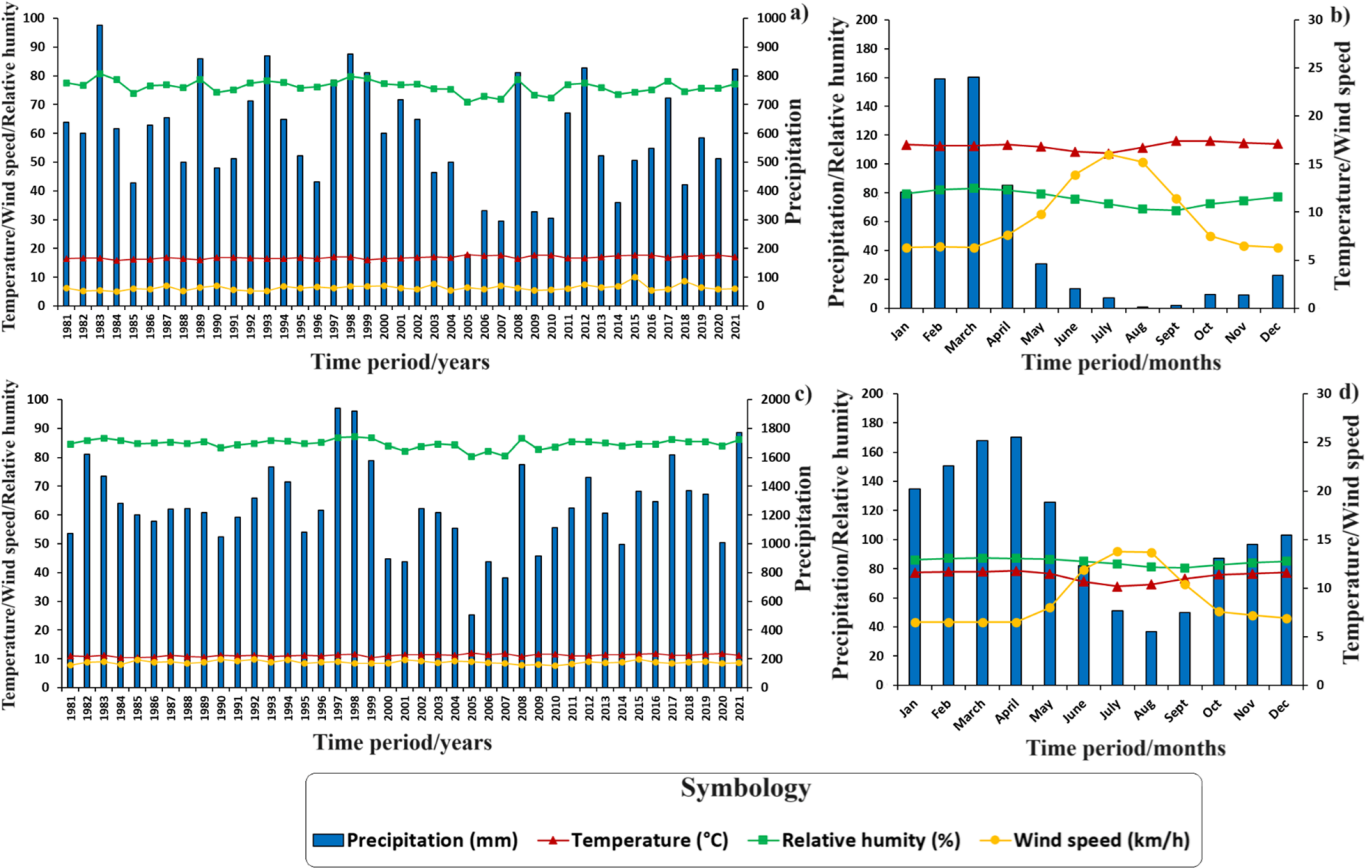
Climographs of the Sierra region, generated with NASA meteorological data (period: 1981–2021). **a** Historical climograph of Sd; **b** Climograph with monthly values of Sd; **c** Historical climograph of SM; and **d** Climograph with monthly values of SM

**Fig. 7:**
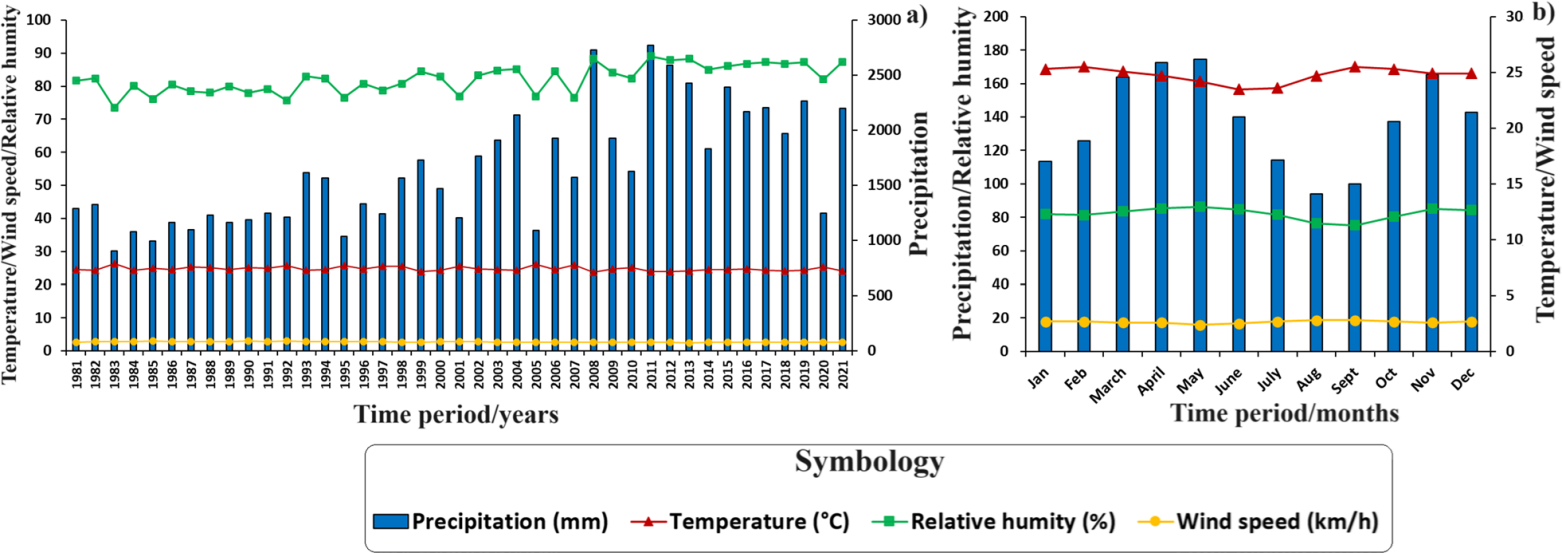
Climographs of the Amazon region, generated with NASA meteorological data (period: 1981–2021). **a** Historical climograph of 7dJ; **b** Climograph with monthly values of 7dJ

Understanding climatic behavior in the regions studied is fundamental in the context of the human use of fire in Ecuador and its potential impact on the occurrence of wildfires. This aspect is essential to understand how climatic variations influence the frequency and severity of wildfires. In addition, the assessment of key variables such as precipitation (mm), relative humidity (%), wind speed (km/h), and temperature (°C) during the wildfire season is vital to develop effective management and prevention strategies. Detailed data provide an in-depth understanding of the climatic dynamics that directly impact the spread of wildfires, especially in coast and mountainous areas. In this way, drier months can be identified as periods of higher wildfire risk, highlighting the need to implement appropriate control and management measures during these critical junctures. In this context, Figs. 5, 6, and 7 present historical and annual climographs from 1981 to 2021 for the examined parishes within Ecuador’s natural landscapes. Figure 5a and c sheds light on interannual climate variability in the Al and SB coast regions, with precipitation emerging as the most variable factor. Notable precipitation shifts include Al’s wettest years in 1983, 1997, and 1998, juxtaposed with the drier spells in 1985, 2005, and 2007. SB mirrors a similar trend. Figure 5b and d delineates monthly climographs, revealing wetter months (December to May) characterized by heightened precipitation, increased relative humidity, and decreased wind speed. Conversely, the six driest months (June to November) signify the wildfire season, marked by diminished precipitation, reduced relative humidity, and elevated wind speeds. Temperature fluctuations are minimal in Al and SB, averaging approximately 25.9 °C and 25.1 °C during the dry and rainy seasons, respectively.

Figure 6a and c depicts interannual climatic variability in the Sd and SM Sierra parishes, with precipitation being the most variable factor. Sd experienced its wettest years in1983, 1998, and 2021, mirroring a similar trend in SM. Figure 3b highlights wetter months from December to May, while Fig. 3d showcases wetter months from October to June, characterized by increased precipitation, heightened relative humidity, and reduced wind speed. Conversely, the driest months mark the wildfire season. Temperature changes are minimal Sd and SM.

In the Amazon parish 7dJ, interannual rainfall variability is evident, with the wettest years observed in 2008, 2011, and 2012, and the driest years recorded in 1983, 1995, and 2005. Rainfall occurs throughout the year, with the lowest precipitation levels noted in August and September, conducive to traditional burning practices. During these months, relative humidity decreases, and temperature increases, with wind speed remainings constant throughout the year.

### Wildfires severity

**Fig. 8:**
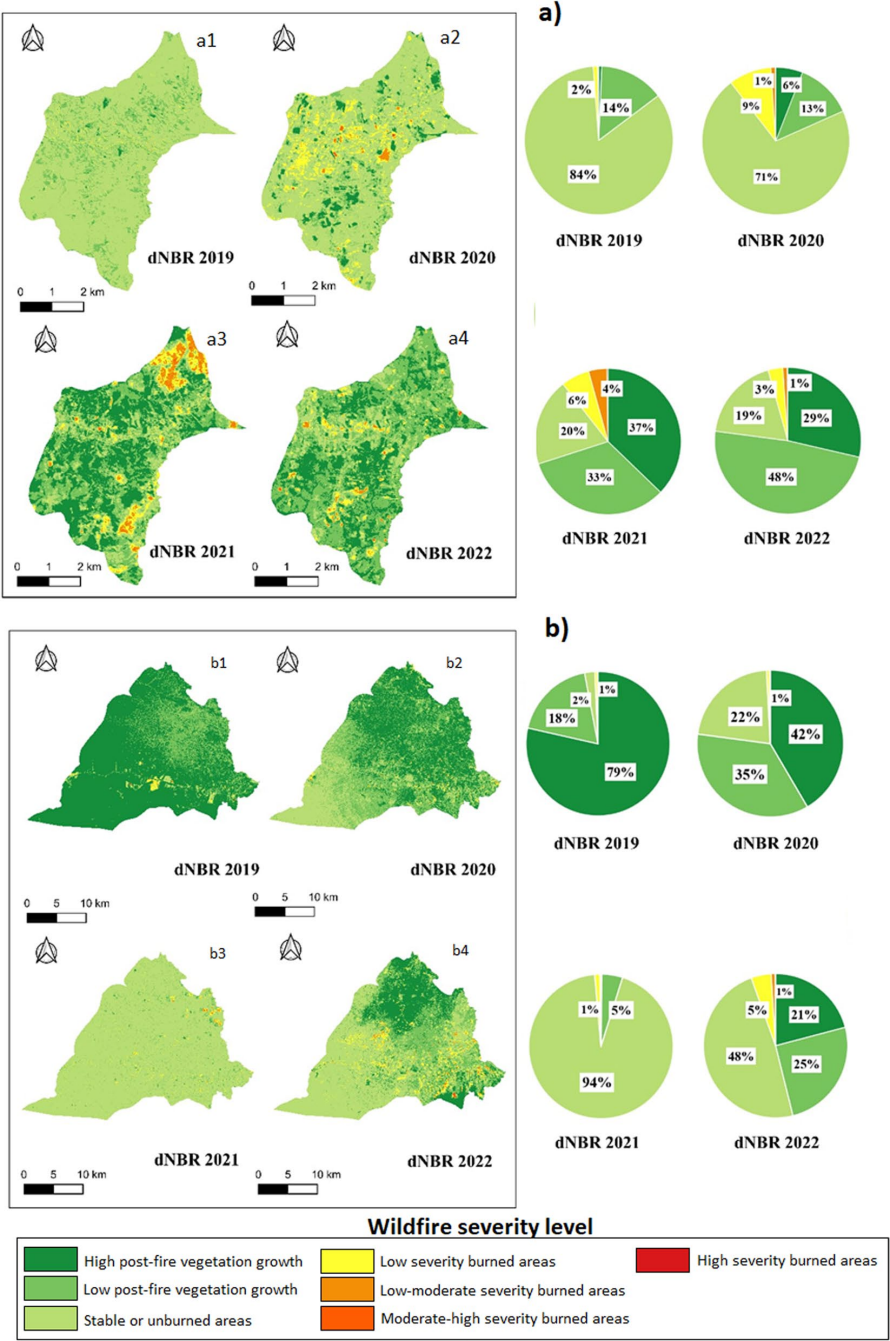
Wildfire severity maps in two parishes of the coast region. **a** Wildfire severity at the scale of Alhajuela parish: **a1** severity for 2019; **a2** severity for 2020; **a3** severity for 2021; and **a4** severity for 2022. **b** Wildfire severity at the Simon Bolivar parish scale: **b1** severity for 2019; **b2** severity for 2020; **b3** severity for 2021; and **b4** severity for 2022. The percentage of fire severity for each contrasted year is presented below

**Fig. 9:**
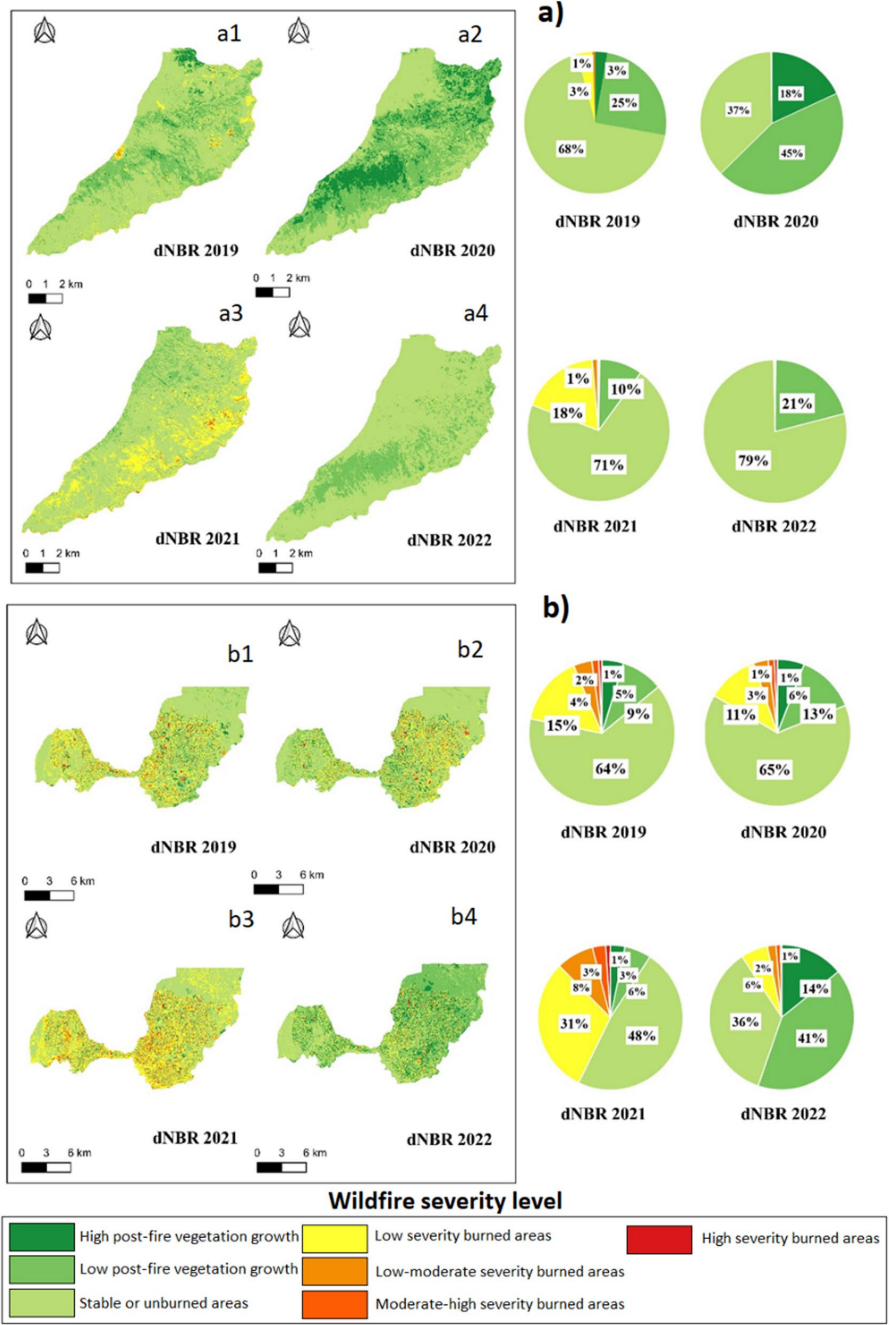
Wildfire severity maps for two parishes in the Sierra region. **a** Severity of wildfires at the Susudel parish scale: **a1** severity for 2019; **a2** severity for 2020; **a3** severity for 2021; and **a4** severity for 2022. **b** Severity of wildfires at the San Miguel parish scale: **b1** severity for 2019; **b2** severity for 2020; **b3** severity for 2021; and **b4** severity for 2022. Below is the percentage of fire severity for each year contrasted

**Fig. 10:**
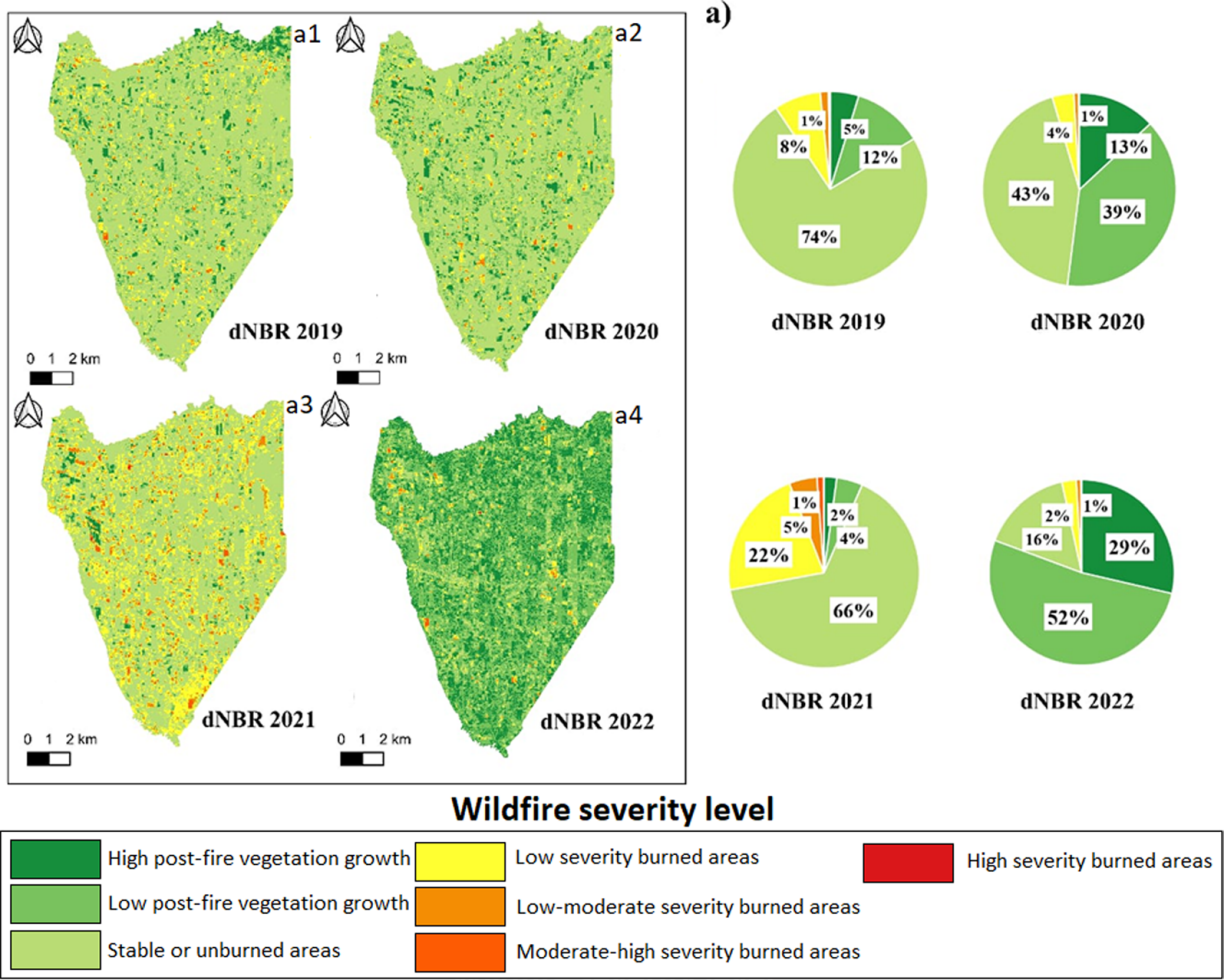
Wildfire severity maps for 7 de Julio parish in the Amazon region. **a** Severity of wildfires: **a1** severity for 2019; **a2** severity for 2020; **a3** severity for 2021; and **a4** severity for 2022. The percent fire severity for each contrasting year is shown below

The remote sensing method has provided valuable insights into fire activity across Ecuador's three natural regions over the last four years, revealing variable intensities ranging from low to moderate-low severity (Figs. 8, 9, and 10). In the coast region, specifically in Al, the year 2019 witnessed low-severity fires affecting approximately 2% of the territory, with 84% remaining stable or unburned. In 2020, low-severity fires increased (9%), along with areas affected by moderate-low-severity fires (1%). In 2021, low-severity fires affected 6% of the territory, allowing vegetation recovery with a notable growth of 37%. In 2022, low severity fires persisted (3%) with a 29% growth. Overall, SB experienced low-severity wildfires, with a notable vegetation recovery rate reaching 99% in 2021. Although there was a slight decrease in 2022 (94%), this data indicates a surprising vegetation regeneration through despite the presence of fires.

In the Sierra region, Sd observed 3% low-severity fires in 2019, with 1% presenting moderate-low severity. No wildfires were recorded in 2020, possibly due to the COVID-19 pandemic lockdown, showing signs of recovery. In 2021, 18% of the fires were low severity, with 1% moderate-low severity. No wildfires were reported in 2022. In SM, there was a variety in the severity of wildfires, with 15% low-severity fires in 2019, decreasing to 6% in 2022.

In 7dJ, variability in the severity of wildfires was observed, with 8% low-severity fires in 2019 and a significant increase in 2021 (22% low severity). In 2022, the number of fires decreased (2% low severity).

### Integration of traditional knowledge and meteorological data for fire calendar development

**Fig. 11:**
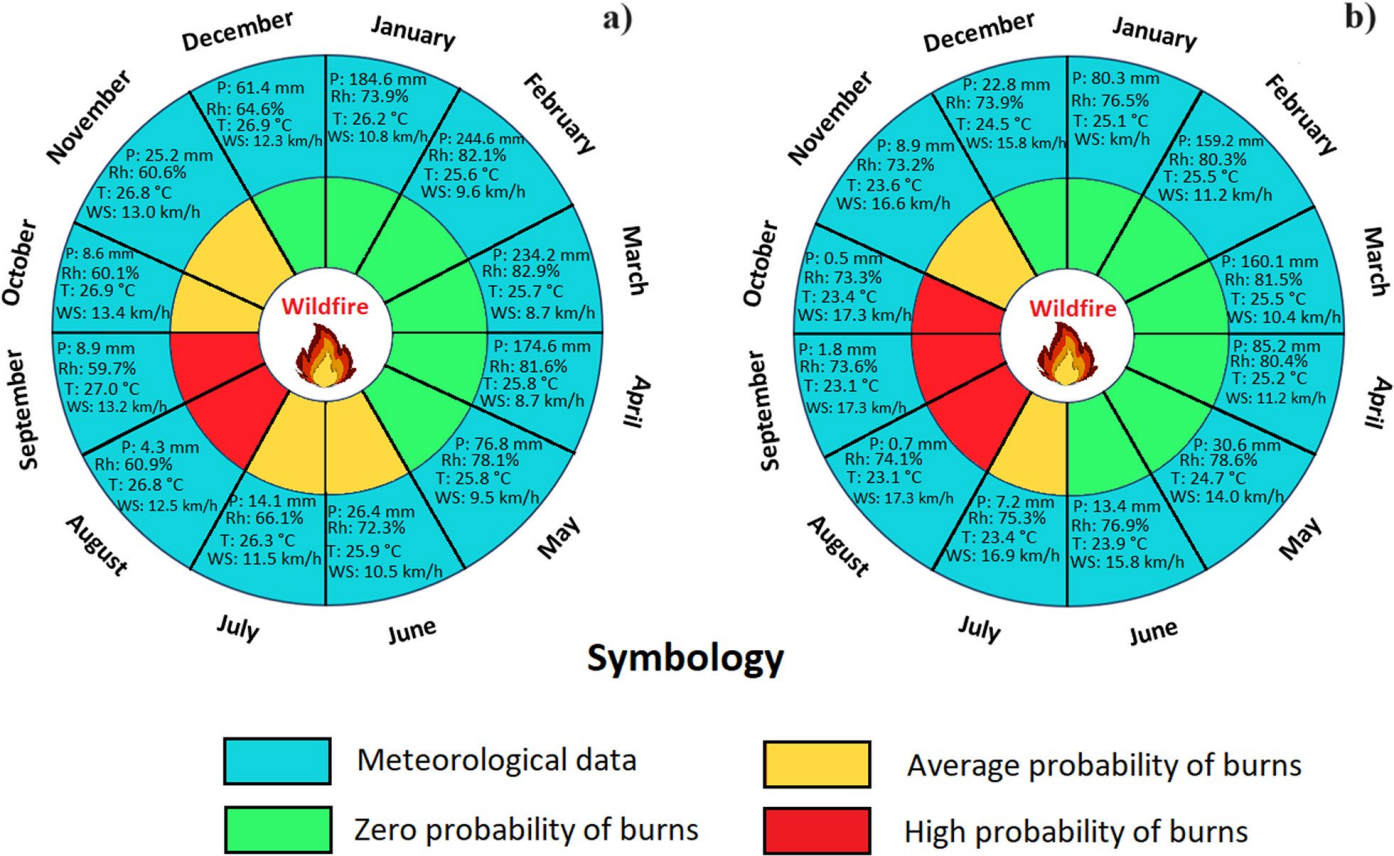
Traditional burning calendar based on local knowledge of farmers on the Ecuadorian coast, compared with NASA meteorological data. **a** Al Parish and **b** SB Parish. Monthly averages of temperature (T), relative humidity (Rh), precipitation (P) (sum), and wind speed (Ws) analyzed over the four years studied were used

**Fig. 12:**
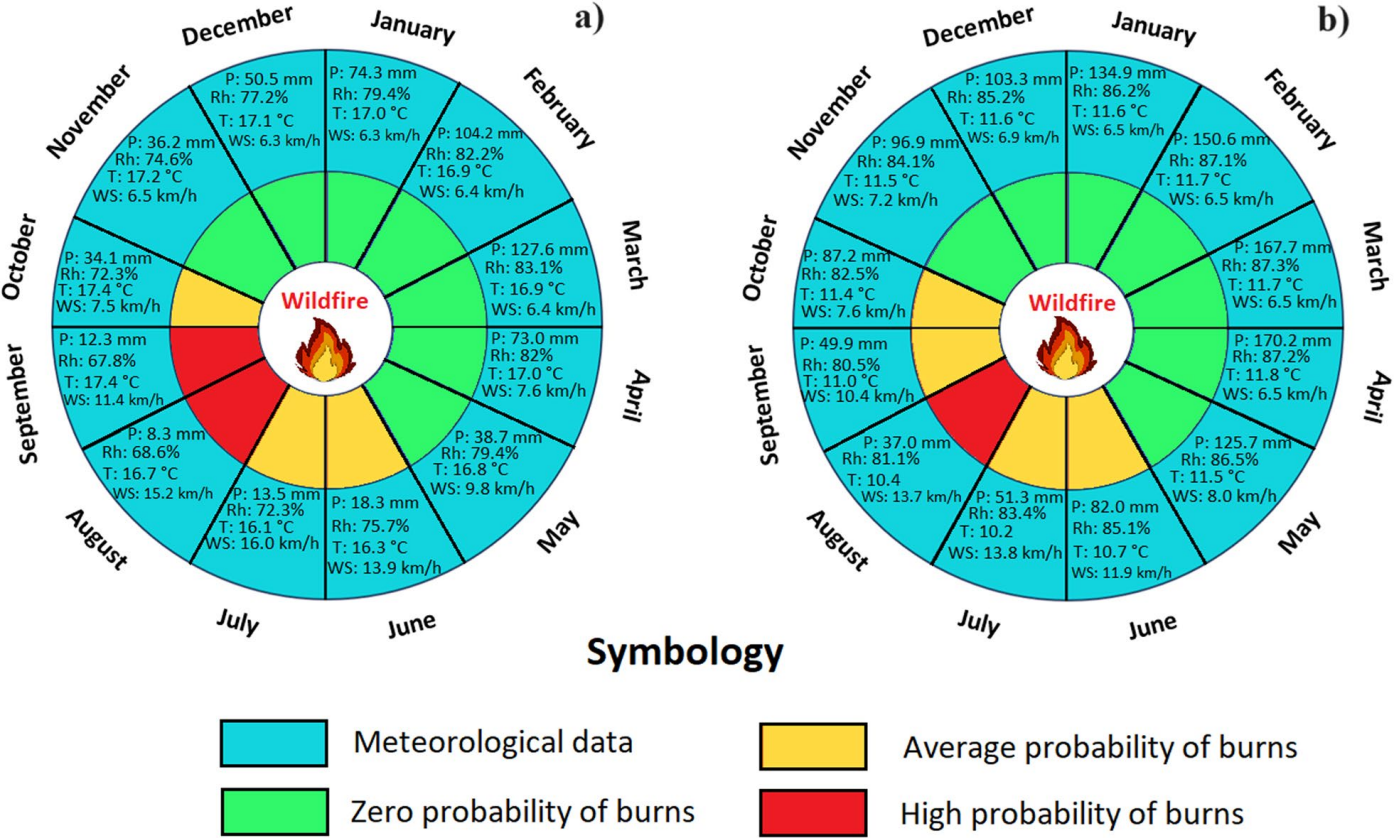
Traditional burning calendar based on local knowledge of farmers on the Ecuadorian Sierra, compared with NASA meteorological data. **a** Sd Parish and **b** SM Parish. Monthly averages of temperature (T), relative humidity (Rh), precipitation (P) (sum), and wind speed (Ws) analyzed over the four years studied were used

**Fig. 13:**
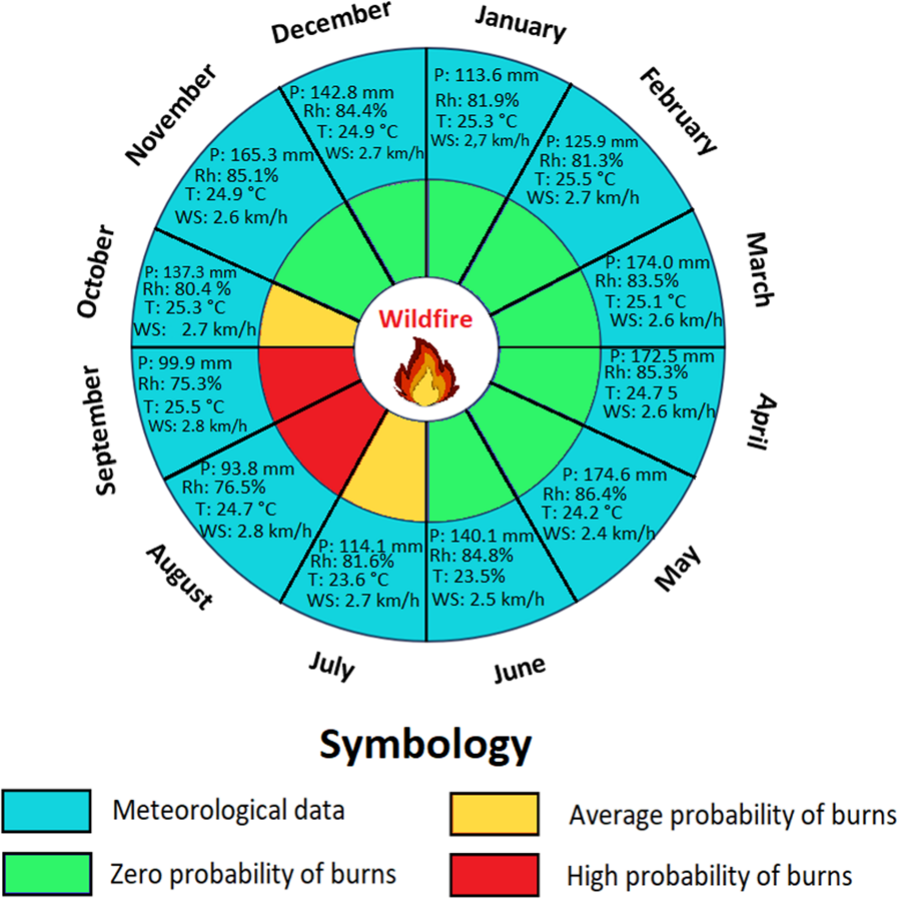
Traditional burning calendar based on local knowledge of farmers on the Ecuadorian Amazon, compared with NASA meteorological data. **a** 7 de Julio. Monthly averages of temperature (T), relative humidity (Rh), precipitation (P) (sum), and wind speed (Ws) analyzed over the four years studied were used

Figures 11, 12, and 13 illustrate three climatic phases in the regions of Ecuador that guide farmers in implementing burns. Along the coast region (Al), a first phase spanning six months, from December to May (depicted in light green), exhibits a zero probability of ignition. This period, marked by substantial rainfall and humidity, aligns with NASA climate data, recording 1002.6 mm of precipitation, an average relative humidity of 76.5%, an average temperature of 26.3 °C, and an average wind speed of 10.0 km/h. The second phase, covering July, October, and November (depicted in yellow), entails a moderate probability of ignition, with decreased precipitation and humidity, increased temperature, and wind speed. The phase of high ignition probability in August and September (depicted in red) is the key season for traditional burns, characterized by reduced precipitation (13.2 mm), lower humidity (60.3%), increased temperature (26.9 °C), and a consistent wind speed (12.9 km/h). A comparable pattern is evident in SB parish (Fig. 11b). During the zero-ignition probability months (December to June), recorded values include 551.6 mm precipitation, 78.3% relative humidity, 24.9 °C temperature, and 12.8 km/h wind speed. The second phase, covering July and November, sees reduced precipitation and humidity (16.1 mm and 74.3%, respectively), a temperature decline (23.5 °C), and increased wind speed (16.8 km/h). The phase with the highest ignition probability, August, September, and October, is marked by significantly reduced precipitation (3.0 mm), a relative humidity decrease (73.7%), constant temperature (23.2 °C), and a relative increase in wind speed (17.3 km/h).

In the Sierra region, mirroring the Coast region, the climatic phases identified by interviewees are evident (Fig. 12). In the parish of Sd, from November to May, zero-ignition probability prevails (504.5 mm total precipitation, 79.7% average relative humidity, 17.3 °C average temperature, and 7.0 km/h wind speed). June, July, and October exhibit a moderate ignition probability, featuring reduced precipitation (65.9 mm total), relative humidity (73.4%), and temperature (16.6 °C), but increased wind speed (12.5 km/h). The high ignition probability phase occurs in August and September, marked by decreased precipitation (20.6 mm total) and relative humidity (68.2%), while maintaining a temperature of 17.1 °C and experiencing the highest wind speed at 13.3 km/h. SM presents a similar climatic pattern.

In the Amazon region, a similar pattern is observed, with more pronounced meteorological variables compared to the coast and Sierra regions (Fig. 13). In parish 7dJ, the zero-ignition phase spans from November to July (1208.8 mm total precipitation, 84.1% average relative humidity, 24.8 °C average temperature, and 2.6 km/h wind speed). The moderate ignition phase covers July through October (251.4 mm, 81.0%, 24.8 °C, and 2.7 km/h). However, the phase of high ignition probability occurs in August and September, with decreased precipitation and relative humidity (193.7 mm and 75.9%, respectively), while maintaining temperature and wind speed (25.1 °C and 2.8 km/h, respectively). Interviewees identify this phase as the most effective and consider these months as the favorable season for traditional burning.

## Discussion

In Ecuador, farmers in the three natural regions integrate fire into their ecosystem management practices, seeking their well-being and that of their families, as has been observed in other parts of the world [69]. This is consistent with previous research suggesting that human activities, including fire use, are the main drivers of ecosystem modeling, challenging the idea that only natural and climatic factors are responsible for these changes [2, 3, 5]. A notable example of this correspondence in Ecuador is the work done by is indigenous communities in the western USA, who maintain long-standing burning traditions for natural resource management [70, 71]. For example, the research of Bowcutt [72] and Long et al. [73] reveals that these communities, which carry out low-intensity burning like those in Ecuador, after applying the fire they collect essential foods such as roots, berries, and mushrooms, crucial elements for family subsistence. Lynn et al. [74] and Larson et al. [75] highlight the direct dependence on products derived from fire, underlining their its importance for community subsistence. In addition, low-severity burning is crucial for the regeneration of plants, bryophytes, and lichens [40], contributing to human well-being, and has positive implications for migratory wildlife.

In the specific context of Ecuador, there are distinct differences and similarities in fire management across the three studied regions, shaped by their unique ecological and cultural contexts. On the coast, the primary causes of wildfires are linked to agricultural activities, whereas in the Sierra and the Amazon, they are mainly due to campfires poorly extinguished by campers. Land preparation practices that lead to wildfires also vary by region. In coast areas, deforestation and the burning of vegetation are predominant, while in the Sierra and the Amazon, these activities have a significantly lower incidence. Consequently, the frequency of fires also varies across these regions. On the coast, monthly, quarterly, and annual fires are more common, while in the Sierra and the Amazon, fires occur primarily on an annual and semi-annual basis. In this context, it is possible that coast farmers, by conducting monthly and quarterly burns during the wetter months, are taking advantage of the climatic conditions in this region that are not optimal for the spread of high-severity fires. In contrast, in the Sierra and the Amazon, where the climatic conditions are inherently wetter than on the coast and the burns are conducted in the climatically optimal months, this context may be producing low-severity fires. This traditional knowledge can be crucial for carrying out controlled low-severity burns, thus minimizing the risk of significant damage to the ecosystems in these regions. Additionally, this practice reflects a deep understanding of local climatic dynamics and their influence on fire behavior. Incorporating this type of knowledge into integrated fire management (IFM) plans can be highly beneficial, as it allows for the adaptation of burning strategies to the specific conditions of each region, ensuring sustainability and the protection of ecosystems.

Furthermore, there are similarities among these contrasting areas that explain why there are more low-severity fires in all three regions. This is because farmers apply specific criteria, such as wind speed and direction, and select flat lands for agricultural burning, allowing them to achieve low-severity burns for crops like maize (*Zea mays*) and potatoes (*Solanum tuberosum*).. These differences and similarities, reflected in traditional burning techniques, could be effectively integrated as mentioned earlier into an integrated fire management (IFM) system or burning plans. In support of this idea, Vázquez-Varela et al. [76] advocate for the incorporation of traditional knowledge from local communities into fire management approaches, proposing more effective IFM strategies [77]. Given the positive impacts observed in the studied areas of Ecuador, characterized by regimes of low to moderate-severity fire, it would be important to consider farmers' perspectives on fire use, which could generate innovative proposals for IFM. Therefore, we argue that validating the relevance of incorporating traditional knowledge about fire use in Ecuador's ecosystem management is crucial, as it has been shown that excluding fire (criminalizing its use) in many regions of the world has been associated with the proliferation of mega-fires and the decline of critical species, promoting the presence of competing vegetation [78]. Thus, further research is needed to confirm or challenge the benefits of traditional burning in the studied parishes.

From the perspective of fire ecology, it is essential to consider the optimal months for wildfire occurrence, as meteorological conditions and fire seasons exhibit significant variations in both Ecuador regions and neighboring countries such as Colombia [79]. For instance, in the coast region, during its dry season (classified as fire weather), a relative aridity is observed compared to other areas, with precipitation extending from July to November, averaging less than 25.0 mm per month a relative humidity of 75%. Conversely, the Sierra region experiences more abundant rainfall during its dry season, spanning from June to October, with monthly averages of less than 87.0 mm and a relative humidity of 85%. As for the Amazon region, it presents consistent rainfall during its dry season, from July to October, with monthly averages of less than 140.0 mm and a relative humidity of 81%. Despite these climatic variations across the three studied regions, wildfires tend to occur primarily in August and September, characterized by reduced precipitation and humidity, as well as increased temperature and wind speed (Figs. 5, 6, and 7). These findings align with previous research conducted by Carrión-Paladines et al. [39] and White [80], emphasizing the critical importance of planning comprehensive fire management programs for these months in Ecuador, given their impact on the wildfire cycle.

Additionally, despite climatic variability in Ecuador's natural regions (Coast, Sierra, and Amazon), remote sensing analysis highlights that most wildfires from 2019 to 2022 were of low and moderate-low severity in all studied areas. However, variations in the dimensions of fires, measured by the percentage of areas affected by low and moderate-low severity, are evident among the analyzed years. This variation may be linked to climatic factors such as interannual precipitation variability, indicating dry and wet years [81]. Furthermore, topography and soil characteristics, closely related to the structure and composition of vegetation [82], significantly influence the extent, frequency, and severity of fires. These findings are consistent with Carrión-Paladines et al. [39] and Díaz et al. [30], who observed significant differences in the territory affected by wildfires in southern Ecuador over four years. Overall, this evidence underscores the crucial role of climate variability, topography, soil factors, and vegetation cover in shaping wildfire patterns and severity in the region.

The low and moderate-low severity of fires identified in this study does not necessarily imply a threat to ecosystems, supporting previous research indicating that these fires are natural components in various terrestrial ecosystems [18]. Richter et al. [83] and Moya et al. [84] emphasize adaptations, such as fire-induced germination mechanisms and fire tolerance in plant communities facing low-severity fires, with temporary changes in soil properties. Additionally, Chandra and Bhardwaj [85] point out that these fires, by burning soil organic matter, improve nutrient availability and favor plant regeneration, while Sulwiński et al. [86] find elevated levels of phosphate in areas moderately affected by fires.

While this study suggests the benefits of low-severity fires for ecosystems, their connection to other factors, especially soil disturbances or the conversion of natural forests to agriculture, as observed in the 7 de Julio parish in the Amazon (Fig. 4), could be exerting a significant ecological influence. For instance, the Ecuadorian Amazon faces challenges such as indiscriminate logging for forest conversion to agricultural areas [87], impacting the quality and health of the soil in its primary properties and affecting vegetation cover [88]. Therefore, in line with recent studies like Coppoletta et al. [89], the low fuel load in cultivated areas, as evidenced in this study, may lead to low-severity fires. Hence, conducting further research to verify the benefits or damages of low-severity burns through a comprehensive analysis of soil properties (physical, chemical, and microbiological) is suggested, as traditional burning has been shown to enhance soil fertility and aeration while increasing organic matter [90, 91]. Additionally, we recommend additional studies on the frequency of low-severity burns, as most in the study area are annual. These studies will be essential to discern their impacts, considering that repeated annual burns, including low-severity ones, may reduce soil carbon compared to less frequent burns (biannual, triennial, and quadrennial) [92]. However, we advocate for the integration of traditional knowledge to enhance our understanding of the benefits of low-severity fire on human well-being [30]. This way, it will be possible to contribute to our understanding of the role of fire in Ecuadorian ecosystems and strengthen integrated fire management practices in the country.

On the other hand, we propose the need to implement alternatives in the studied areas to reduce the annual use of fire, avoiding possible negative impacts, as indicated by recent studies [92]. Among the main strategies and alternatives to the use of fire are the adoption of conservation agricultural practices, the implementation of agroforestry and silvopastoral systems, productive diversification and the production of organic fertilizers using crop residues and productive processes, such as those developed in some areas of Ecuador [93].

In addition to that, there is an urgent need to reform Ecuador's legal framework, which lacks essential provisions for traditional burning that affect the study area and other regions of the country. The absence of municipal regulations addressing climatic factors and wildfire severity levels contrasts with regional practices. For example, Chile has incorporated concepts of severity into a new wildfire law, focusing on low-severity fires to reduce dangerous conditions [94]. Similarly, in the USA, fires of different severity levels are evaluated to improve ecosystem performance [95]. It is then necessary to reform fire suppression and prevention policies in Ecuador, since they could be causing ecological damage over time, due to the accumulation of fuel load as studied by Norgaard [96]. However, it is encouraging to see innovative approaches to fire management in Brazil and Venezuela, where indigenous burning practices are being integrated into an intercultural governance framework [14]. These approaches, supported by centuries-old traditional ecological knowledge, have proven their effectiveness in these nations. Therefore, Ecuador should base new fire management programs on a detailed assessment of the local socioecological context, incorporating crucial concepts such as fire climate, severity, and traditional knowledge about fire use [30]. This is because people are the direct users of ecosystems, and they are the ones who use fire. It may be necessary then, that in Ecuador new ordinances be implemented at the municipal level since there is still a need to implement controlled and prescribed burning plans with a participatory approach in the country since in this study, for example, farmers do not use firebreak strips when carrying out their burning. However, these plans must be adapted to the human and environmental needs of each parish studied and in this way develop a comprehensive and efficient fire management system.

Finally, it is important to highlight that in Ecuador, farmers use traditional calendars, to identify the optimal times for conducting their traditional burns. This practice is like those observed in other ecosystems around the world, where various studies have documented the use of seasonal calendars by rural communities, based on worldviews and ecological factors. For example, in Arnhem Land, Australia, the population uses the Yugul Mangi Faiya Kelenda (fire calendar), while in Ecuador, the indigenous Saraguro community employs the Community Fire Calendar, which takes into account the “Veranillo del Niño” (VdN) phenomenon to carry out low-severity burns in the high-Andean páramos [30, 58]. The validation of this community fire calendar with meteorological data, conducted in collaboration with farmers and representatives of parish councils, ensured the relevance and accuracy of the calendar in relation to traditional practices and the specific environmental conditions of each parish. These hybrid calendars, combining traditional knowledge with climatic information, are valuable tools for integrated fire management (IFM), providing a practical and culturally relevant guide for fire management practices in the various study regions in Ecuador.

## Conclusions

This study has shown that the traditional use of fire by farmers in three regions of Ecuador is a crucial tool for the sustainable management of natural resources. Following a traditional burning calendar and ancestral practices, farmers strategically schedule burning during the driest months (generally August to September). These low-severity annual fires offer important benefits to the ecosystem, in line with global research findings that have determined that they promote human well-being. However, to validate the suitability of the annual frequency of fires in Ecuador, long-term studies on the impacts on soil, water, vegetation, and native fauna are recommended. On the other hand, to address the legal loophole associated with the ancestral use of fire, Ecuador should formulate new regulations at the municipal level that cover issues such as fire climate, degrees of severity, and traditional practices. Such legislation would not only offer a new management perspective, as controlled and prescribed burning plans would be implemented, but would also improve the current National Integrated Fire Management Strategy (2021–2025). The knowledge derived from this study has the potential to guide policymakers in developing effective measures that encourage responsible fire use in fire-affected ecosystems in Ecuador.

## Data Availability

The datasets used and/or analyzed during the current study are available from the corresponding author on reasonable request.

## Abbreviations

NBR: Normalized Burn Ratio
dNBR: Difference between pre-fire and post-fire
TKFU: Traditional knowledge of fire use
Al: Alhajuela Parish
SB: Simón Bolívar Parish
Sd: Susudel Parish
SM: San Miguel Parish
7dJ: Siete de Julio Parish
LIF: Level of information fidelity
ICF: Informant's consensus factor
IFM: Integrated fire management
